# Probiotics or synbiotics addition to sows’ diets alters colonic microbiome composition and metabolome profiles of offspring pigs

**DOI:** 10.3389/fmicb.2022.934890

**Published:** 2022-08-17

**Authors:** Qian Zhu, Mingtong Song, Md. Abul Kalam Azad, Yating Cheng, Yating Liu, Yang Liu, François Blachier, Yulong Yin, Xiangfeng Kong

**Affiliations:** ^1^Hunan Provincial Key Laboratory of Animal Nutritional Physiology and Metabolic Process, Key Laboratory of Agro-Ecological Processes in Subtropical Region, National Engineering Laboratory for Pollution Control and Waste Utilization in Livestock and Poultry Production, Institute of Subtropical Agriculture, Chinese Academy of Sciences, Changsha, China; ^2^College of Advanced Agricultural Sciences, University of Chinese Academy of Sciences, Beijing, China; ^3^Université Paris-Saclay, AgroParisTech, INRAE, UMR PNCA, Paris, France; ^4^Research Center of Mini-Pig, Huanjiang Observation and Research Station for Karst Ecosystems, Chinese Academy of Sciences, Huanjiang, China

**Keywords:** bacterial metabolites, Bama mini-pigs, microbiome, probiotics, synbiotics

## Abstract

Little information exists about the effects of maternal probiotics and synbiotics addition on the gut microbiome and metabolome of offspring. The present study evaluated the effects of probiotics or synbiotics addition to sows’ diets on colonic microbiota and their metabolites in offspring using 16S rRNA gene sequencing and metabolome strategy. A total of 64 pregnant Bama mini-pigs were randomly divided into control, antibiotic, probiotics, and synbiotics groups and fed the corresponding experimental diets during pregnancy and lactation. After weaning, two piglets per litter and eight piglets per group were selected and fed a basal diet. The β-diversity analysis showed that the colonic microbiota of offspring had a clear distinction among the four groups at 65 days of age. Maternal probiotics addition increased the Actinobacteria abundance at 65 days of age and Tenericutes and Firmicutes abundances at 95 days of age of offspring compared with the other three groups, whereas maternal antibiotic addition increased Spirochaetes and Proteobacteria abundances at 95 days of age of offspring compared with the other three groups. Metabolomic analysis showed that colonic metabolites were different between the groups, regardless of the days of age. Furthermore, both PICRUSt2 and enrichment analysis of metabolic pathways showed that maternal probiotics and synbiotics addition affected metabolism of carbohydrate, amino acid, cofactors and vitamins in the colonic microbiota. Compared with the control group, the colonic concentration of indole decreased and skatole increased in the probiotics group, whereas indole increased and skatole decreased in the synbiotics group. Maternal probiotics addition increased the colonic concentrations of acetate and butyrate at 65 and 125 days of age, whereas probiotics and synbiotics addition decreased short-chain fatty acids concentrations at 95 days of age. In addition, the colonic concentrations of putrescine, cadaverine, 1,7-heptanediamine, and spermidine were increased in the antibiotic, probiotics, and synbiotics groups compared with the control group at 95 days of age. The correlation analysis showed that *Gemmiger*, *Roseburia*, and *Faecalibacterium* abundances were positively correlated with acetate, propionate, and butyrate concentrations; *Gemmiger*, *Blautia*, and *Faecalibacterium* were positively correlated with putrescine and spermidine; and *Faecalibacterium*, *Blautia*, *Clostridium*, and *Streptococcus* were positively correlated with (R)-3-hydroxybutyric acid. Collectively, these findings suggest that probiotics and synbiotics addition to sows’ diets exerts effects on offspring pigs by altering gut microbiota composition and their metabolites. The potential beneficial effect on gut health is discussed.

## Introduction

Microbiota harbored in the mammalian gut not only have the major function of harvesting undigested or not fully digested dietary compounds, but also influence a range of metabolic, developmental, and physiological processes of the host (Xiao et al., 2020). The gut microbiota exert their effects notably by fermenting dietary ingredients to produce various bioactive compounds (Schroeder and Bäckhed, 2016). These metabolites signal to the intestinal mucosa, and after absorption, distant organs in the body, thus enabling enteric bacteria to connect to host metabolism by regulating several metabolic pathways and impacting the physiological and pathological status of the host (Koh et al., 2016; Blachier et al., 2017). Gut microbiota live in a symbiotic interaction with the host and co-exist in dynamic equilibrium when contributing to host physiology (Monteagudo-Mera et al., 2016). In recent years, more attention has been paid to the colonization, composition, and function of intestinal microbes. Microbial colonization in the mammalian gut occurs at the very first life stage and undergoes drastic changes during early childhood (Wang et al., 2016), which represents an important driver for the development and maturation of the gut and likely contributes significantly to the long-term health of the host (Korpela et al., 2018). In addition, bacteria transmission from the mother to the neonate through direct contact with maternal microbiota during birth and breast milk during lactation also seems to influence the gut colonization of the infant, with potential health consequences (Sanz, 2011). Considering that the gut microbiota of piglets is mainly derived from the sows’ intestinal strains (Ferretti et al., 2018), the study of the regulation of the maternal intestinal microecology deserves attention.

It has been documented that probiotics and synbiotics may represent an effective strategy to improve the gut microbiota in animals and humans. The health-promoting potentials of probiotics include maintenance of gut homeostasis, alienating pathogens, enhancing the nutrient bioavailability, and stimulating and modulating host immune system (Xavier-Santos et al., 2019). A balanced microbiota is an indispensable constituent of a healthy gut, and probiotics can correct the microbiota imbalance in the gut on some occasions, and improve the overall health of the host (Patil et al., 2019). Probiotics and synbiotics also have gained considerable attention concerning their beneficial effects on livestock performance and health. Dietary probiotics supplementation can improve gut health and nutrient digestibility and thus benefit nutrient utilization and growth performance of pigs. Previous studies have demonstrated that probiotics improved the reproductive performance of sows and the growth performance of neonatal piglets by improving intestinal microbiota (Hayakawa et al., 2016). Moreover, synbiotics may stimulate the growth of beneficial microbiota, and enhance the production of beneficial bacterial metabolites like short-chain fatty acids (SCFAs) in sows, while decreasing the production of deleterious metabolites, such effects being possibly associated with improved growth performance and gut microbiota balance in piglets (Śliżewska and Chlebicz, 2019; Girard et al., 2021). Furthermore, the effects of dietary probiotics and synbiotics may mainly target the cecum and colon of pigs, where an abundant and diverse microbial population is harbored (Liao and Nyachoti, 2017). For example, probiotics and prebiotics are conducive to the increase of beneficial microbiota through the growth and production of their metabolites in the host (Sanders et al., 2019). Prebiotics has beneficial effects on the ecological and genetic stability of gut microbiota (Ma et al., 2020a). Moreover, synbiotics may play beneficial roles in the gut microbiota of pigs (Chlebicz-Wojcik and Slizewska, 2020).

Our previous studies showed that dietary synbiotics supplementation could alter the composition of gut microbiota in pregnant and lactating sows and improve colonic microbiota composition and metabolic activity in suckling piglets (Ma et al., 2020b,c). Moreover, maternal probiotics and synbiotics supplementation may improve the antioxidant capacity, mitochondrial function, and immune response of weaned piglets by modifying the gut microbiota (Wang et al., 2021a,b). Furthermore, it can be hypothesized that maternal probiotics and synbiotics addition can improve feed intake and meat quality by altering the metabolism and gene expression related to the meat quality of offspring. However, the long-term effects of probiotics and synbiotics addition to sows’ diets on the colonic microbiota and metabolites in offspring pigs are poorly known. Based on the foregoing, the present study hypothesized that the long-term effects of maternal probiotics and synbiotics addition might regulate the colonic microbiota and metabolome of offspring pigs. Bama mini-pigs are a famous local miniature pig breed in China, and different meat processing methods for this pig breed have different slaughter weight requirements. A previous study has reported that 7.5–10 kg mini-pigs are generally used for roasting pork, whereas pigs with heavier body weights are used for processing bacon (Cai et al., 2021). This study was conducted using Bama mini-pigs to determine the effects of probiotics and synbiotics addition to sows’ diets on colonic microbiome and metabolome of offspring pigs during different time points (65, 95, and 125 days of age) after weaning and explored the correlation between microbiota and their metabolites. These findings will provide a basis for the application of probiotics and synbiotics in mother-offspring integration.

## Materials and methods

### Animals, diets, and treatments

This study was conducted at the mini-pig experimental base of Goat Chong, Shimen Town, Changde City, Hunan Province, China. A total of 64 pregnant Bama mini-pigs with parities of 3–5 and initial body weight (BW) of 92.60 ± 11.76 kg were selected and randomly divided into four groups with 16 sows (pens) per group. The treatment groups included the control group (fed antibiotic-free basal diet), antibiotic group (SA, 50 g/t virginiamycin with the basal diet), probiotics group (SP, 200 mL/d probiotics mixture per animal with the basal diet), and synbiotics group [SS, 500 g xylo-oligosaccharides (XOS) per ton diet + 200 mL/d probiotics mixture per animal with the basal diet]. The probiotics mixture was provided by Hunan Lifeng Biotechnology Co., Ltd. (Changsha, Hunan, China), and contained *Lactobacillus plantarum* B90 (CGMCC1.12934) ≥ 1 × 10^8^ CFU/mL and *Saccharomyces cerevisiae* P11 (CGMCC2.3854) ≥ 0.2 × 10^8^ CFU/mL. The XOS (≥ 35%) was provided by Shandong Longlive Biotechnology Co., Ltd. (Shandong, China) and contained xylobiose (55%), xylotriose (25%), xylotetraose (10%), xylopentose (5%), xylohexaose (3%), and xyloheptaose (2%), which met the feed additive of XOS recommended requirements (GB/T23747-2009). The supplemented probiotics mixture was mixed with the feed before feeding the sows, and XOS was added during feed production. The doses of the probiotics and synbiotics were as recommended by the manufacturers and referred to the previous studies (Tan et al., 2016; Ma et al., 2020c).

The sows were housed individually in gestation crates (2.2 × 0.6 m) from day 1 to day 105 of pregnancy, transferred to farrowing crates (2.2 × 1.8 m) on day 106 of pregnancy, and housed until weaning. Creep feed was provided to the suckling piglets from 7 to 28 days of age. After weaning, at 28 days of age, two piglets close to the average BW per litter were selected and transferred to the nursery house for the subsequent feeding trial. After one week of adaption, four piglets from two litters in the same group were merged into one pen. There were eight pens (replicates) and 32 piglets per group. A total of 128 piglets were fed the basal diet for the remaining days of the trial. The composition and nutrient levels of basal diets for sows and piglets are presented in Supplementary Tables 1, 2, respectively. Feeding and management were performed according to the standard operations of commercial pig farms.

### Sample collection

At 65, 95, and 125 days of age, the offspring pigs from each group were fasted for 12 h and weighed, and then one pig per pen (a total of eight pigs per group) was selected and euthanized under commercial conditions using electrical stunning (120 V, 200 HZ) and exsanguination. Then the pigs were dissected and the head, legs, tail, and viscera were removed. The colonic contents were sampled into sterile centrifuge tubes and immediately stored at −20°C until further analysis for indole, skatole, SCFAs, and bioamines, and stored at −80°C until further analysis for microbiota and metabolites.

### Deoxyribonucleic acid extraction, Illumina MiSeq sequencing, data processing, and analysis

Total genomic deoxyribonucleic acid (DNA) from the individual samples of colonic contents was extracted using the Fast DNA SPIN extraction kits (MP Biomedicals, Santa Ana, CA, United States) according to the manufacturer’s instructions. The DNA concentration of each sample was quantified using the NanoDrop ND-1000 spectrophotometer (Thermo Fisher Scientific, Waltham, MA, United States), and the resulting polymerase chain reaction (PCR) products were separated using agarose gel electrophoresis. The genes of all microbial 16S rRNA in the hypervariable regions of V3−V4 were amplified by PCR using a universal forward primer F (5′-ACTCCTACGGGAGGCAGCA-3′) and a reverse primer R (5′-GGACTACHVGGGTWTCTAAT-3′). The PCR thermal cycle conditions were as below: 2 min initial denaturation at 98°C, 25 cycles of 15 s at 98°C, 30 s annealing at 55°C, and 30 s elongation at 72°C, and a final extension at 72°C for 5 min. The PCR components include: 5 μL of Q5 reaction buffer (5×), 5 μL of Q5 High-Fidelity GC buffer (5×), 0.25 μL of Q5 High-Fidelity DNA Polymerase (5 U/μL), 2 μL (2.5 mM) of dNTPs, 1 μL (10 μM) each of forward and reverse primers, 2 μL of DNA template, and 8.75 μL of ddH_2_O. The PCR amplicons were purified using the Agencourt AMPure Beads (Beckman Coulter, Indianapolis, IN, United States) and quantified with the PicoGreen dsDNA Assay Kit (Invitrogen, Carlsbad, CA, United States), according to the manufacturer’s instructions. The purified amplicons were pooled in equimolar from each sample and paired-end sequenced (2 × 300) using the NovaSeq 6000 SP Reagent Kit (500 cycles) on an Illumina MiSeq platform (Illumina, San Diego, CA, United States), according to the standard protocols by Shanghai Personal Biotechnology Co., Ltd. (Shanghai, China).

The raw sequence data generated from 16S rRNA NovaSeq sequencing were demultiplexed and quality-filtered using quantitative insights into microbial ecology (QIIME2; version 2019.4) with slight modification according to the official tutorials.^1^ Sequences were quality filtered, denoised, merged, and chimera removed using the DADA2 plugin (Callahan et al., 2016). After quality control and filtering chimeras, non-singleton amplicon sequence variants (ASVs) were aligned with mafft (Katoh et al., 2002) and used to construct a phylogeny with fasttree2 (Price et al., 2010). Taxonomy was assigned to ASVs in the Greengenes database using a classify-sklearn naïve Bayes taxonomy classifier in the feature-classifier plugin against the Greengenes 13_8 99% operational taxonomic units (OTUs) reference sequences (Bokulich et al., 2018). The α-diversity indices, including rarefaction analysis, Chao1, Observed species, Shannon, Simpson, and Pielou’s evenness, were estimated using the diversity plugin. Non-metric multidimensional scaling (NMDS) plots based on the Bray-Curtis distance metric were used to visualize differences in microbial community composition among the groups. Analysis of similarity (ANOSIM) for multivariate data was performed using the “vegan” package in R.^2^ The partial least square discriminant analysis (PLS-DA) was also performed using the R package to visualize the differences in microbial community composition. The Kruskal-Wallis test was used to identify statistically different microbial taxa at phylum and genus levels among the four groups. The abundance of different microbiota and the Kyoto Encyclopedia of Genes and Genomes (KEGG) pathways were classified using the linear discriminant analysis (LDA) effect size algorithm if the logarithmic LDA values of bacteria exceeded 2.0 using the online procedure of Galaxy.^3^ The 16S rRNA sequencing data obtained in this study are deposited in the NCBI Sequence Read Archive (SRA) with under the accession number PRJNA825463^4^.

### Colonic metabolome and data analysis

The colonic contents were put into the 2-mL EP tube with two steel balls and homogenized by the tissue grinder. The homogenized samples (100 mg) were vortexed for 30 s with 0.6 mL methanol (including internal standard), then grinded, and centrifuged at 12,000 × *g* for 10 min at 4°C to obtain the supernatant. The supernatant was filtered through 0.22 μM membrane to obtain the prepared samples for liquid chromatography-tandem mass spectrometry (LC-MS). The details were as previously described (Chen et al., 2017). The quality control samples were obtained by mixing a small and equal volume of each experimental sample and injected at regular intervals to monitor the stability of the analysis. The LC-MS analysis was performed on Vanquish Ultrahigh-performance LC System (Thermo Fisher Scientific, Waltham, MA, United States) coupled with an Orbitrap Q Exactive series mass spectrometer (Thermo Fisher Scientific). The raw data files were converted into mzXML format by Proteowizard software (v3.0.8789) and then processed by the XCMS software^5^ for peaks identification, filtration, and alignment. The metabolites were identified by comparison with the internal library using the mass-to-charge ratio (m/z), retention time, and chromatographic data. The internal standard was used for data QC (reproducibility), and metabolic features in which the relative standard deviation (RSD) of QC > 30% was discarded. The metabolite annotation was performed with the Compound Discoverer program and referenced to the mzCloud database,^6^ as well as MetDNA, BioDeepDB, and MoNA.^7^

Principal components analysis (PCA) using an unsupervised method was applied to obtain an overview of the metabolic data, general clustering, and trends. Orthogonal projections to latent structures discriminate analysis (OPLS-DA) was used for statistical analysis to determine the global metabolic changes among the four groups. Variable importance in projection (VIP) was calculated in the OPLS-DA model. The fitting validity and projective ability of the selected OPLS-DA model were assessed by the parameters *R*^2^Y and *Q*^2^Y, respectively. Discriminating metabolites among the four groups were identified using a statistically significant VIP threshold of value (VIP ≥ 1) and further validated by univariate analysis of variance (ANOVA) analysis (*P* ≤ 0.05). The significantly different abundant metabolites screened from untargeted metabolomics were imported into the MetaboAnalyst 5.0 and KEGG databases^8^ to perform pathway analysis. Heatmaps were constructed using Euclidian distances and complete linkage grouping with the pheatmap package in R (see text footnote 2). The correlation between the different metabolites was evaluated with the Spearman’s rank correlation test using the R package.

### Colonic short-chain fatty acids, indole, skatole, and bioamines analysis

The SCFAs concentrations in colonic contents were determined by gas chromatography (Agilent 7890A, Agilent Inc., Palo Alto, CA, United States) according to the method described in previous studies (Ji et al., 2018). Indole, skatole, and bioamines concentrations in colonic contents were measured using high-performance liquid chromatography (Agilent 1290, Agilent Inc., Palo Alto, CA, United States) as described previously (Hu et al., 2019).

### Statistical analysis

One-way ANOVA (SPSS 25.0; IBM Inc., Chicago, IL, United States) and Tukey *post-hoc* test were used to analyze the data of colonic metabolites and visualized using GraphPad Prism version 8.0 (GraphPad Software, San Diego, CA, United States). The level of significance was set at *P*-value < 0.05. The analysis of the correlation between metabolites and microbiota abundance at the genus level was determined with the Spearman’s rank correlation test using the R package (see text footnote 2).

## Results

### Microbial diversity in colonic contents of the offspring pigs

**FIGURE 1:**
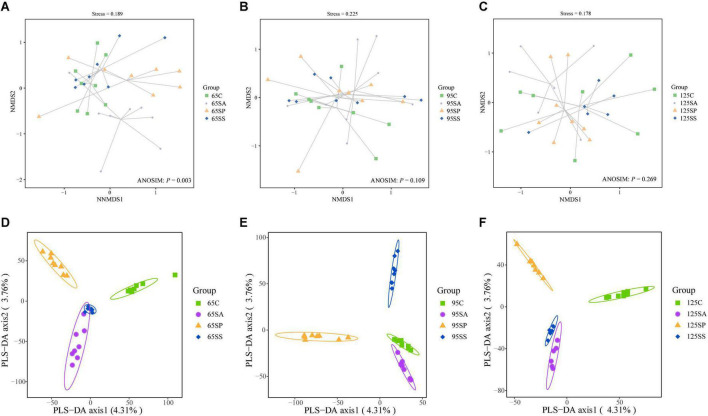
Non-metric multidimensional scaling (NMDS) ordination plots based on the Bray-Curtis distance metric **(A–C)** and partial least square discriminant analysis (PLS-DA) plots **(D–F)** of the colonic microbiota community of offspring pigs at 65, 95, and 125 days of age. 65C, 95C, and 125C, sow fed with basal diet; 65SA, 95SA, and 125SA, sow fed with antibiotic; 65SP, 95SP, and 125SP, sow fed with probiotics; 65SS, 95SS, and 125SS, sow fed with synbiotics.

The rarefaction curves show that the sampling in each group provided sufficient OTU coverage (Supplementary Figure 1). The statistical estimates of α-diversity from each sample at a genetic distance of 3% are presented in Supplementary Table 3. No effects were observed on any indices due to probiotics and synbiotics addition to sows’ diets (Supplementary Table 3), including OTUs, richness estimators (Chao1 and Observed species), diversity indices (Shannon and Simpson), and evenness index (Pielou’s). Then the β-diversity analysis was conducted to measure the dissimilarity of the microbial communities. The NMDS ordination plot based on the Bray-Curtis distance metric showed that the microbial communities in colonic contents were clearly separated at 65 days of age (Figure 1A; ANOSIM, *P* = 0.003). However, there were not clearly separated among the four groups at 95 and 125 days of age (Figures 1B,C; ANOSIM, *P* > 0.05). Furthermore, the PLS-DA indicated that the microbial communities in colonic contents at 65, 95, and 125 days of age of offspring pigs were clearly separated and clustered into distinct groups (Figures 1D–F).

### Microbiota structure in colonic contents of the offspring pigs

**FIGURE 2:**
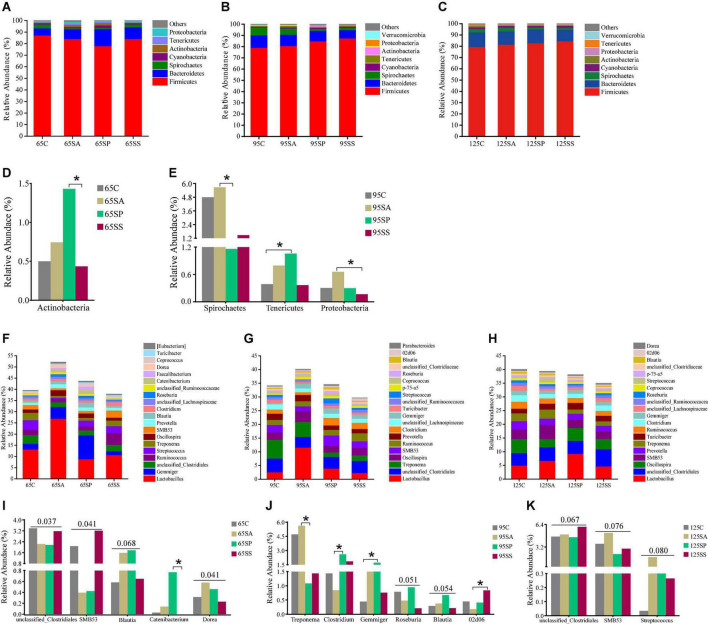
Relative abundance of colonic microbiota at phylum level (relative abundance > 0.1%) **(A–C)** and genus level (the top 20 genera) **(F–H)** of offspring pigs. The significant changes of phyla at 65 **(D)** and 95 **(E)** days of age and genera at 65 **(I)**, 95 **(J)**, and 125 **(K)** days of age are presented. The values are expressed as the median. Statistical differences are calculated by the Kruskal-Wallis test: Significance is considered at a *P*-value < 0.05 and the tendency is considered at a *P*-value between 0.05 and 0.1. Asterisk represents *P* < 0.05. 65C, 95C, and 125C, sow fed with basal diet; 65SA, 95SA, and 125SA, sow fed with antibiotic; 65SP, 95SP, and 125SP, sow fed with probiotics; 65SS, 95SS, and 125SS, sow fed with synbiotics.

At the phylum level, the dominant microbiota were Firmicutes, Bacteroidetes, Spirochaetes, and Cyanobacteria at 65, 95, and 125 days of age (Figures 2A–C), while other phyla were present at very low relative abundances. At 65 days of age, these four phyla accounted for 97.84, 94.56, 95.95, and 97.87% of the reads for offspring pigs from the four groups, respectively (Figure 2A). At 95 days of age, these four phyla accounted for 97.81, 97.41, 96.84, and 97.98% of the reads for offspring pigs from the four groups, respectively (Figure 2B). At 125 days of age, these four phyla accounted for 97.03, 97.82, 97.92, and 97.85% of the reads for offspring pigs from the four groups, respectively (Figure 2C). At the genus level, the ten most dominant genera were *Lactobacillus*, *Gemmiger*, *unclassified_Clostridiales*, *Ruminococcus*, *Streptococcus*, *Treponema*, *Oscillospira*, *SMB53*, *Prevotella*, and *Blautia* at 65 days of age (Figure 2F); *Lactobacillus*, *unclassified_Clostridiales*, *Treponema*, *Oscillospira*, *SMB53*, *Ruminococcus*, *Prevotella*, *Clostridium*, *unclassified_Lachnospiraceae*, and *Gemmiger* at 95 days of age (Figure 2G); and *Lactobacillus*, *unclassified_Clostridiales*, *Oscillospira*, *SMB53*, *Prevotella*, *Treponema*, *Turicibacter*, *Ruminococcus*, *Clostridium, unclassified_Lachnospiraceae*, and *Gemmiger* at 125 days of age (Figure 2H). These results indicated that *Lactobacillus* was the most dominant genus in different treatment groups and at different growth stages. *Lactobacillus*, *Gemmiger*, *unclassified_Clostridiales*, *Ruminococcus*, *Treponema*, *Oscillospira*, *SMB53*, and *Prevotella* were the common genera at 65, 95, and 125 days of age.

Significant differences of microbiota at phylum and genus levels in colonic contents among the four groups at different stages were further identified using Kruskal-Wallis analysis. At 65 days of age, the relative abundance of Actinobacteria was increased (*P* < 0.05) in offspring pigs from the SP group compared with the SS group (Figure 2D). At 95 days of age, the relative abundances of Spirochaetes from the SP group and Proteobacteria from the SS group in offspring pigs were decreased (*P* < 0.05) compared with the SA group (Figure 2E); the relative abundance of Tenericutes was increased (*P* < 0.05) in offspring pigs from the SP group (Figure 2E) compared with the control group. At the genus level, there was an increasing trend in the relative abundances of *Blautia* (*P* = 0.068) and *Dorea* (*P* = 0.041) in the SA and SP groups at 65 days of age, however, *unclassified_Clostridiales* (*P* = 0.037) and *SMB53* (*P* = 0.041) were decreased (Figure 2I) compared with the control and SS groups. The relative abundance of *Catenibacterium* in the SP group was increased (*P* < 0.05) compared with the SS group at 65 days of age (Figure 2I). At 95 days of age, the relative abundance of *Treponema* in the SP group was decreased (*P* < 0.05), whereas that of *Clostridium* was increased (*P* < 0.05) compared with the SA group. Compared with the SA group, the relative abundance of *02d06* was increased (*P* < 0.05) in the SS group. There was a remarkable increase (*P* < 0.05) in the relative abundance of *Gemmiger* in the SP group compared with the control group. The relative abundances of *Roseburia* (*P* = 0.051) and *Blautia* (*P* = 0.054) in the SP group had an increasing trend compared with the other three groups (Figure 2J). At 125 days of age, there was an increasing trend in the relative abundance of *unclassified_Clostridiales* (*P* = 0.067) in the SS group compared with the other three groups, as well as *SMB53* (*P* = 0.076) and *Streptococcus* (*P* = 0.080) in the SA group (Figure 2K).

### The linear discriminant analysis effect size analysis of different microbiota in colonic contents of the offspring pigs

The clustering heat map with the abundance of the genus with the top 50 average abundance was drawn to analyze the trend of species abundance distribution in each treatment group. The results indicated that there were different enriched genera among the four groups at 65, 95, and 125 days of age (Supplementary Figure 2).

**FIGURE 3:**
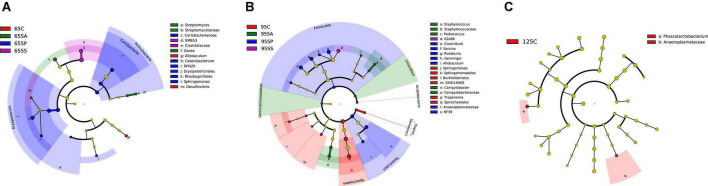
Different enrichment of microbiota in colonic contents of offspring pigs. Analysis of taxonomic abundance using linear discriminant analysis effect size (LEfSe analysis) (LDA score ≥ 2, *P* < 0.05) in colonic contents of offspring pigs at 65 **(A)**, 95 **(B)**, and 125 **(C)** days of age. The cladogram shows the microbial species with a significant difference between the experimental groups and the control group. 65C, 95C, and 125C, sow fed with basal diet; 65SA, 95SA, and 125SA, sow fed with antibiotic; 65SP, 95SP, and 125SP, sow fed with probiotics; 65SS, 95SS, and 125SS, sow fed with synbiotics.

At the phylum level, the LEfSe analysis revealed that Actinobacteria was enriched in the SP group at 65 days of age (Figure 3A). In addition, there were relatively higher Spirochaetes and [Thermi] abundances in the control group, Acidobacteria, Gemmatimonadetes, and Chloroflexi in the SA group, and Tenericutes and Firmicutes in the SP group at 95 days of age (Figure 3B). At the genus level, the LEfSe analysis indicated that *Allobaculum* and *Desulfovibrio* were enriched in the control group, *Streptomyces* and *Dorea* were enriched in the SA group, *Sphingomonas*, *Catenibactrium*, and *RFN20* were enriched in the SP group, and *SMB53* was enriched in the SS group at 65 days of age (Figure 3A). Moreover, *Treponema* and *Sphingomonas* were enriched in the control group, *Pediococcus*, *Campylobacter*, and *Staphylococcus* were enriched in the SA group, *Clostridium*, *Gemmiger*, *Roseburia*, *Allobaculum*, and *Sarcina* were enriched in the SP group, and *02d06* was enriched in the SS group at 95 days of age (Figure 3B). The relative abundance of *Phascolatobacerium* was enriched in the control group than in the other three groups at 125 days of age (Figure 3C).

### Predicted microbiota functions in colonic contents of the offspring pigs

**FIGURE 4:**
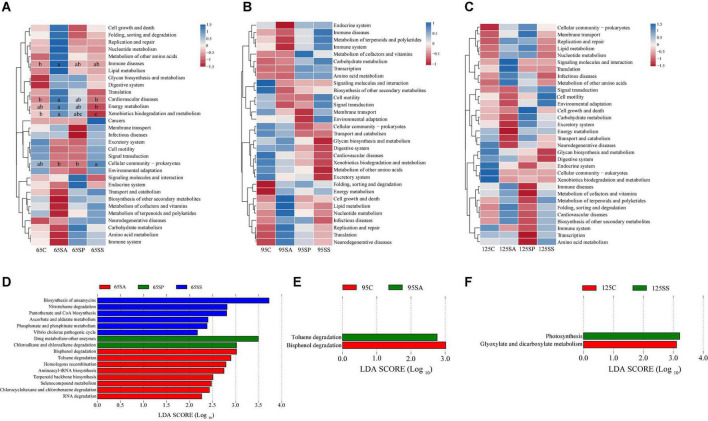
Predicted function of colonic microbiota of offspring pigs between the experimental groups and the control group. The heatmap showed that the comparisons of microbiota pathways among different treatment groups at 65 **(A)**, 95 **(B)**, and 125 **(C)** days of age at level 2 using Kruskal-Wallis test. The LEfSe histogram showed that the different metabolic pathways among different groups at 65 **(D)**, 95 **(E)**, and 125 **(F)** days of age at level 3. Different lowercase letters in the same row were significantly different (*P* < 0.05). 65C, 95C, and 125C, sow fed with basal diet; 65SA, 95SA, and 125SA, sow fed with antibiotic; 65SP, 95SP, and 125SP, sow fed with probiotics; 65SS, 95SS, and 125SS, sow fed with synbiotics.

To understand the microbial metabolic function of the colonic contents, a PICRUSt2 approach was used to predict the KEGG pathway composition of the microbial communities. As shown in Supplementary Figure 3, at level 1, approximately 76.43, 75.68, and 75.89% of the pathways were affiliated with metabolism; 14.80, 14.73, and 14.78% of pathways were involved with genetic information processing; and 2.84, 2.70, and 2.75% of pathways belonged to environmental information processing at 65, 95, and 125 days of age, respectively. At level 2, a total of 31, 32, and 32 KEGG pathways were identified in the microbiota of colonic contents at 65, 95, and 125 days of age, respectively. The majority of these pathways were associated with carbohydrate metabolism, amino acid metabolism, metabolism of cofactors and vitamins, metabolism of terpenoids and polyketides, metabolism of other amino acids, replication and repair, as well as lipid and energy metabolism (Supplementary Figure 3). Then, the differences in functional pathways between different groups were analyzed at level 2 (Figures 4A–C). At 65 days of age, the relative abundances of several pathways were higher in the SA group, including immune disease, cardiovascular disease, energy metabolism, and xenobiotics biodegradation and metabolism, while cellular community-prokaryotes displayed lower relative abundance in the SA group (Figure 4A). There was no significant difference in the functional pathways at 95 and 125 days of age (Figures 4B,C). In addition, the composition of pathways was further analyzed at level 3, identifying 16, 2, and 2 significantly enriched pathways at 65, 95, and 125 days of age, respectively (Figures 4D–F). At 65 days of age, a total of eight pathways (including bisphenol degradation, toluene degradation, homologous recombination, aminoacyl-tRNA biosynthesis, terpenoid backbone biosynthesis, selenocompound metabolism, chlorocyclohexane and chlorobenzene degradation, and RNA degradation) were significantly enriched in the SA group, two pathways (including drug metabolism-other enzymes and chloroalkane and chloroalkene degradation) were significantly enriched in the SP group, and six pathways (including biosynthesis of ansamycins, nitrotoluene degradation, pantothenate and CoA biosynthesis, ascorbate and aldarate metabolism, phosphonate and phosphinate metabolism, and *Vibrio cholerae* pathogenic cycle) were significantly enriched in the SS group (Figure 4D). At 95 days of age, bisphenol degradation was significantly enriched in the control group and toluene degradation was significantly enriched in the SA group (Figure 4E). At 125 days of age, glyoxylate and dicarboxylate metabolism was significantly enriched in the control group and the pathway involved in photosynthesis was significantly enriched in the SS group (Figure 4F).

### Metabolome analysis in the colonic contents of the offspring pigs

**FIGURE 5:**
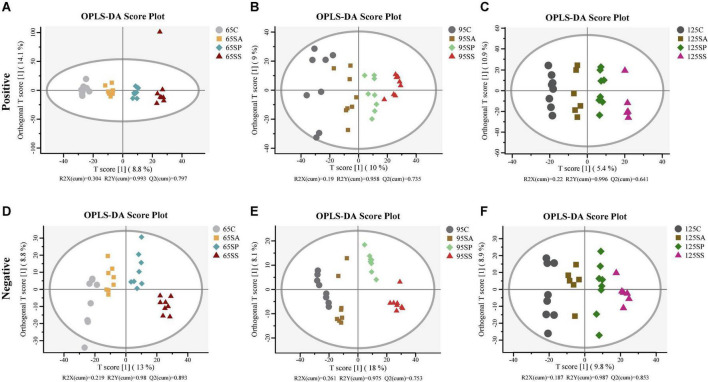
Orthogonal partial least squares discriminant analysis (OPLS-DA) plots based on the metabolites in colonic contents of offspring pigs. The OPLS-DA plots of the metabolites in colonic contents in positive **(A–C)** and negative **(D–F)** modes at 65, 95, and 125 days of age among the four groups. 65C, 95C, and 125C, sow fed with basal diet; 65SA, 95SA, and 125SA, sow fed with antibiotic; 65SP, 95SP, and 125SP, sow fed with probiotics; 65SS, 95SS, and 125SS, sow fed with synbiotics.

The PCA was conducted to visualize the differences in colonic metabolite composition of the offspring pigs in the four groups at different days of age (Supplementary Figure 4). At 65, 95, and 125 days of age, the metabolite composition in offspring pigs was more different among the four groups in the negative model than that in the positive model. To investigate the specific metabolites associated with maternal probiotics and synbiotics addition, the OPLS-DA was carried out, and this analysis was able to appropriately categorize all samples among different groups (Figure 5). The best-fitted OPLS-DA model was selected and validated by a cross-validation of all candidate models using a 200-cycle permutation test (Supplementary Figure 5). These results suggest that the changes occurred in the colonic metabolome of offspring pigs due to maternal probiotics and synbiotics addition.

**FIGURE 6:**
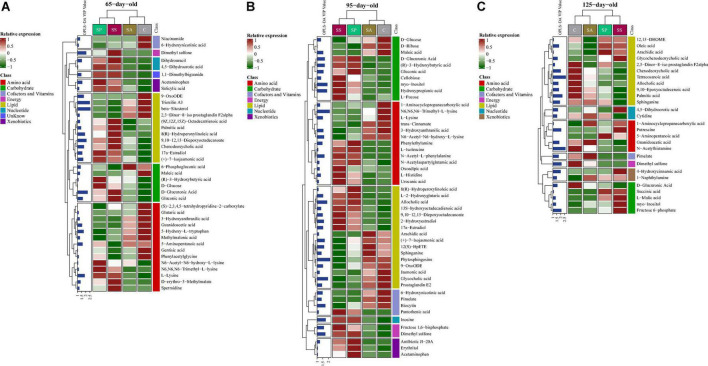
Analysis of different metabolites in colonic contents of offspring pigs. The heatmap of different metabolites at 65 **(A)**, 95 **(B)**, and 125 **(C)** days of age. 65C, 95C, and 125C, sow fed with basal diet; 65SA, 95SA, and 125SA, sow fed with antibiotic; 65SP, 95SP, and 125SP, sow fed with probiotics; 65SS, 95SS, and 125SS, sow fed with synbiotics.

A total of 182 metabolites were identified. Moreover, 39, 49, and 27 metabolites were significantly altered among the four groups at 65, 95, and 125 days of age, respectively (Figure 6). These different metabolites were mainly related to amino acids, carbohydrate, and lipid metabolism. The proportions of these three metabolites were 35.90, 15.38, and 28.21% at 65 days of age (Supplementary Figure 6A), 26.53, 20.41, and 32.65% at 95 days of age (Supplementary Figure 6B), and 18.52, 18.52, and 40.74% at 125 days of age (Supplementary Figure 6C), respectively. The different metabolic patterns in the different experimental groups are shown in Figure 6. When the VIP ≥ 2, there were five (including beta-sitosterol, trioxilin A3, D-glucuronic acid, 9,10–12,13-diepoxyoctadecanoate, and dimethyl sulfone), two (including phytosphingosine and N6,N6,N6-trimethyl-L-lysine), and eight (including tetracosanoic acid, N-acetylhistamine, palmitic acid, dimethyl sulfone, fructose 6-phosphate, putrescine, chenodeoxycholic acid, and 4-hydroxycinnamic acid) different metabolites at 65, 95, and 125 days of age, respectively.

**FIGURE 7:**
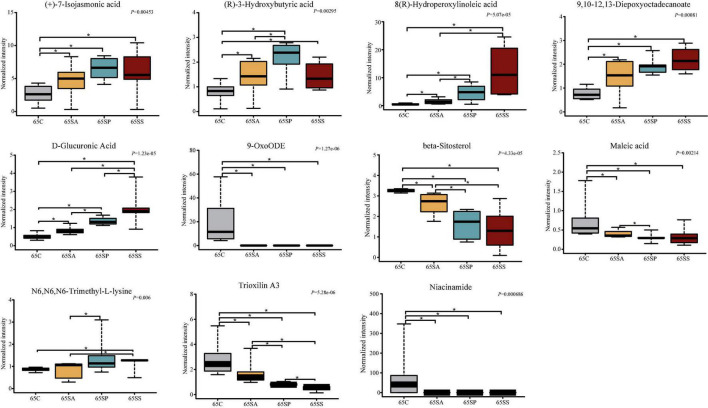
The box plots of different metabolites at 65 days of age. Asterisk represents *P* < 0.05. 65C, sow fed with basal diet; 65SA, sow fed with antibiotic; 65SP, sow fed with probiotics; 65SS, sow fed with synbiotics.

**FIGURE 8:**
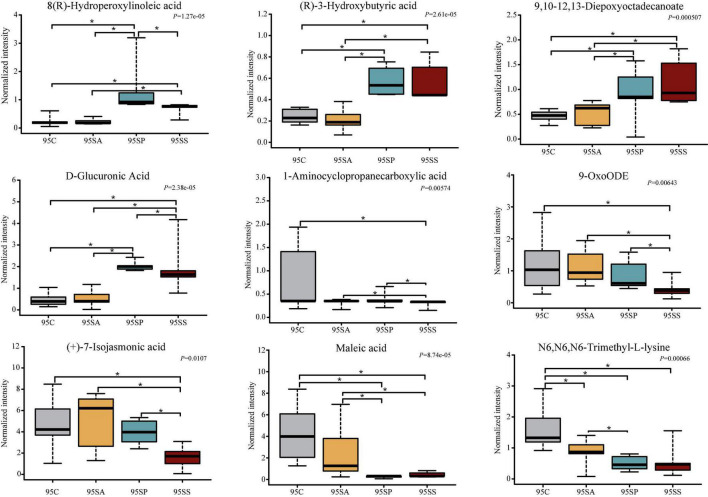
The box plots of different metabolites at 95 days of age. Asterisk represents *P* < 0.05. 95C, sow fed with basal diet; 95SA, sow fed with antibiotic; 95SP, sow fed with probiotics; 95SS, sow fed with synbiotics.

**FIGURE 9:**
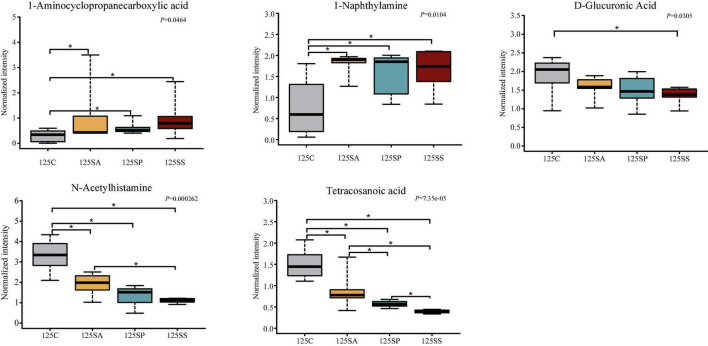
The box plots of different metabolites at 125 days of age. Asterisk represents *P* < 0.05. 125C, sow fed with basal diet; 125SA, sow fed with antibiotic; 125SP, sow fed with probiotics; 125SS, sow fed with synbiotics.

To further identify the pivotal metabolites, a pairwise comparison between different treatment groups was conducted. There were 18 common metabolites at three different days of age (Supplementary Figures 6D–F). Combining the results of the pairwise comparison and ANOVA analysis, there were 11 (Figure 7), 9 (Figure 8), and 5 (Figure 9) metabolites were identified at 65, 95, and 125 days of age, respectively. At 65 days of age, the normalized intensity of (+)-7-isojasmonic acid, (R)-3-hydroxybutyric acid, 8(R)-hydroperoxylinoleic acid, 9,10-12,13-diepoxyoctadecanoate, and D-glucuronic acid was increased (*P* < 0.05) in the SA, SP and SS groups, whereas that of 9-oxoODE, beta-sitosterol, maleic acid, trioxilin A3, and niacinamide was decreased (*P* < 0.05) compared with the control group (Figure 7). The normalized intensity of N6,N6,N6-trimethyl-L-lysine in the SP group was higher (*P* < 0.05) than in the SA group, as well as that in the SS group than in the control and SA groups (Figure 7). At 95 days of age, the normalized intensity of 8(R)-hydroperoxylinoleic acid, (R)-3-hydroxybutyric acid, 9,10-12,13-diepoxyoctadecanoate, and D-glucuronic acid was increased (*P* < 0.05) in the SP and SS groups, while that of maleic acid and N6,N6,N6-trimethyl-L-lysine was decreased (*P* < 0.05) compared with the control group (Figure 8). The normalized intensity of 1-aminocyclopropanecarboxylic acid in the control group was higher (*P* < 0.05) among the four groups (Figure 8). In addition, the normalized intensity of 9-oxoODE and (+)-7-isojasmonic acid in the SS group was decreased (*P* < 0.05) compared with the other three groups (Figure 8). At 125 days of age, the normalized intensity of 1-aminocyclopropanecarboxylic acid and 1-naphthylamine was increased (*P* < 0.05), whereas N-acetylhistamine and tetracosanoic acid was decreased (*P* < 0.05) in the SA, SP, and SS groups compared with the control group (Figure 9). Furthermore, the normalized intensity of D-glucuronic acid in the SS group was lower (*P* < 0.05) than in the control group (Figure 9). In addition, the correlations between the metabolites at 65, 95, and 125 days of age were consistent with the variation trend of the normalized intensity of these metabolites in Figures 7–9, as well as Supplementary Figure 7.

**FIGURE 10:**
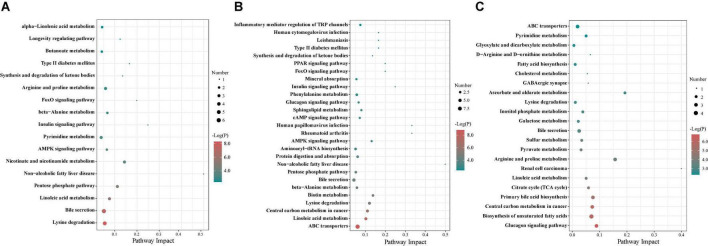
The bubble chart of enrichment analysis for metabolic pathways in colonic contents of offspring pigs at 65 **(A)**, 95 **(B)**, and 125 **(C)** days of age. The manipulated metabolic pathways are based on the analysis of different metabolites in colonic contents of offspring pigs among different groups following the KEGG pathway database. The metabolome view shows all matched pathways according to the *P*-values from the pathway enrichment analysis and impact values from the topology analysis. The node colors varied from green to red, indicating that the metabolites have different levels of significance.

To further investigate metabolic pathways involved in these different metabolites, a differential metabolite pathway analysis was conducted. As shown in Figure 10, several metabolic pathways were affected by maternal probiotics and synbiotics addition. At 65 days of age, the different metabolic pathways were lysine degradation, non-alcoholic fatty liver disease, insulin signaling pathway, nicotinate and nicotinamide metabolism, and pentose phosphate pathway (Figure 10A). At 95 days of age, the primary metabolic pathways were ABC transporters, non-alcoholic fatty liver disease, rheumatoid arthritis, human papillomavirus infection, biotin metabolism, AMPK signaling pathway, lysine degradation, central carbon metabolism in cancer, and linoleic acid metabolism (Figure 10B), and those at 125 days of age were renal cell carcinoma, arginine and proline metabolism, and glucagon signaling pathway (Figure 10C).

### The concentrations of indole, skatole, short-chain fatty acids, and bioamines in colonic contents of the offspring pigs

**FIGURE 11:**
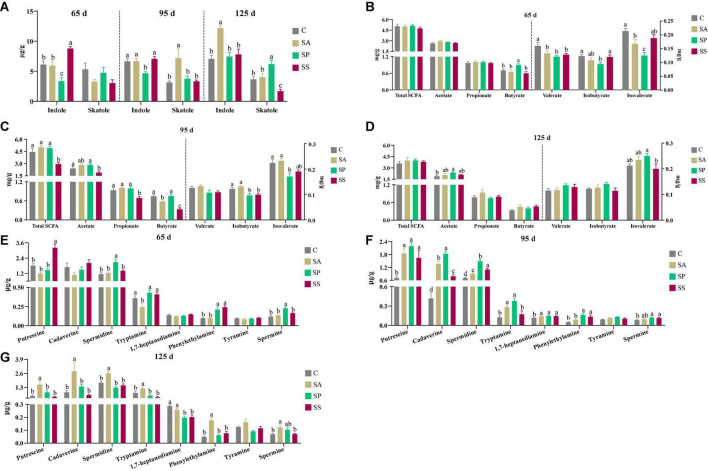
Concentrations of indole, skatole, short-chain fatty acids (SCFAs), and bioamines in colonic contents of offspring pigs at 65, 95, and 125 days of age. Concentrations of indole and skatole in colonic contents at 65, 95, and 125 days of age **(A)**; concentrations of SCFAs in colonic contents at 65, 95, and 125 days of age **(B–D)**, respectively; and concentrations of bioamines in colonic contents at 65, 95, and 125 days of age **(E–G**), respectively. Different superscript letters mean a significant difference (*P* < 0.05). 65 d, 65 days of age; 95 d, 95 days of age; 125 d, 125 days of age; C, sow fed with basal diet; SA, sow fed with antibiotic; SP, sow fed with probiotics; SS, sow fed with synbiotics.

As shown in Figure 11A, the indole concentration was lower (*P* < 0.05) in the SP group at 65 and 95 days of age, whereas that was higher (*P* < 0.05) in the SS group at 65 days of age, as well as that in the SA group at 125 days of age, compared with the other groups. The skatole concentration was higher (*P* < 0.05) in the SA group at 95 days of age, as well as that in the SP group at 125 days of age, whereas that was lower (*P* < 0.05) in the SS group, compared with the other groups.

As shown in Figure 11B, at 65 days of age, the butyrate concentration was higher and isovalerate concentration was lower in the SP group compared with the other groups, whereas isobutyrate concentration was lower in the SP group compared with the control and SS groups (*P* < 0.05). In addition, the valerate concentration in the SA, SP, and SS groups was decreased (*P* < 0.05) compared with the control group. As shown in Figure 11C, at 95 days of age, the concentrations of propionate, butyrate, and total SCFA were lower (*P* < 0.05) in the SS group compared with the other groups; the concentration of acetate was lower (*P* < 0.05) in the SS group than in the control group, whereas isobutyrate in the SP and SS groups and isovalerate in the SP group were lower (*P* < 0.05) compared with the control and SA groups. As shown in Figure 11D, at 125 days of age, the acetate concentration was increased (*P* < 0.05) in the SP group compared with the control group; isovalerate concentration was increased (*P* < 0.05) in the SP group compared with the SS group. As shown in Figure 11E, at 65 days of age, the putrescine concentration was higher (*P* < 0.05) in the SS group, as well as spermidine and spermine in the SP group, compared with the other groups. In addition, the phenylethylamine concentration was increased (*P* < 0.05) in the SP and SS groups compared with the control and SA groups. As shown in Figure 11F, at 95 days of age, the concentrations of putrescine, cadaverine, spermidine, spermine, and 1,7-heptanediamine were increased (*P* < 0.05) in the SA, SP, and SS groups compared with the control group. The phenylethylamine concentration was increased (*P* < 0.05) in the SP and SS groups compared with the control and SA groups. Furthermore, the tryptamine concentration was increased (*P* < 0.05) in the SA and SP groups compared with the control and SS groups. As shown in Figure 11G, at 125 days of age, the concentrations of putrescine, cadaverine spermidine, tryptamine, and phenylethylamine were higher (*P* < 0.05) in the SA group than the other three groups, whereas the spermine concentration was higher (*P* < 0.05) in the SA group compared than the control and SS groups. The 1,7-heptanediamine concentration was decreased (*P* < 0.05) in the SP and SS groups compared with the control and SA groups.

### Correlation between metabolites and microbiota in colonic contents of the offspring pigs

**FIGURE 12:**
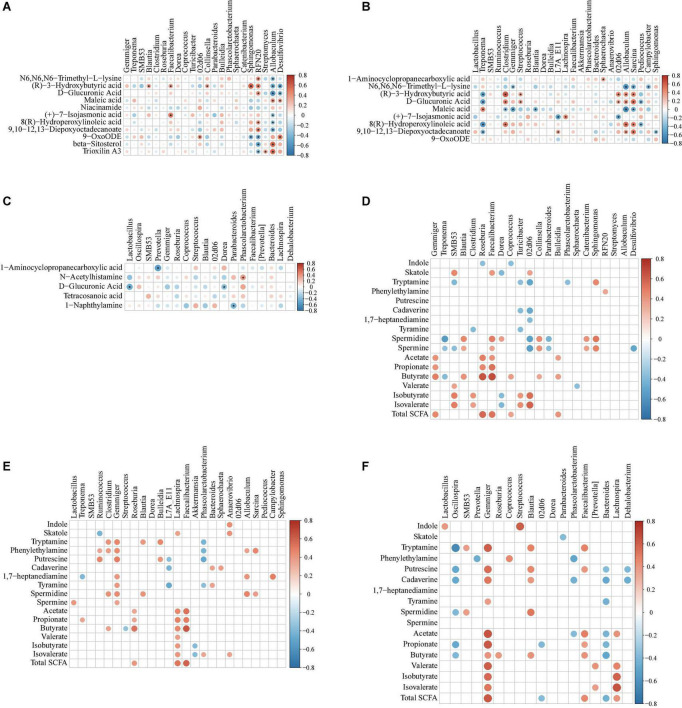
Spearman correlation analysis of different microbiota and metabolites [from metabolomic analysis, **(A–C)** indole, skatole, SCFAs, and bioamine, **(D–F)**] in colonic contents of offspring pigs at 65, 95, and 125 days of age, respectively. The red represents positive correlation while the blue represents negative correlation. Asterisk **(A–C)** and red or blue circle **(D–F)** represents *P*-value < 0.05.

To explore the functional correlation between changes in the colonic microbiota and metabolites, Spearman’s correlation analysis was generated by calculating Spearman’s correlation coefficient among the microbial composition affected by the dietary treatments (at the genus level, adjusted *P* < 0.05) and metabolites (Figure 12). A clear significant correlation (*P* < 0.05) was identified between the changes in the colonic microbiome and metabolome. The correlation analysis revealed that fewer metabolites from the metabolome were correlated with the colonic microbiota, whereas more metabolites (including indole, skatole, SCFAs, and bioamines) were correlated with the colonic microbiota (Figure 12).

As shown in Figure 12A, at 65 days of age, the positive correlation (*P* < 0.05) included between (R)-3-hydroxybutyric acid with *Blautia*; (R)-3-hydroxybutyric acid and (+)-7-isojasmonic acid with *Faecalibacterium*; 9-OxoODE with *02d06*; (R)-3-hydroxybutyric acid with *Collinsella* and *Sphingomonas*; N6,N6,N6-trimethyl-L-lysine, (R)-3-hydroxybutyric acid, D-glucuronic acid, 8(R)-hydroperoxylinoleic acid, and 9,10-12,13-diepoxyoctadecanoate with *RFN20*; maleic acid, beta-sitosterol, and trioxilin A3 with *Allobaculum*; maleic acid and 9-OxoODE with *Desulfovibrio*. In addition, the negative correlation (*P* < 0.05) included between 9-OxoODE with *Sphingomonas*; 9-OxoODE, beta-sitosterol, and trioxilin A3 with *RFN20*; N6,N6,N6-trimethyl-L-lysine, (R)-3-hydroxybutyric acid, D-glucuronic acid, (+)-7-isojasmonic acid, 8(R)-hydroperoxylinoleic acid, and 9,10-12,13-diepoxyoctadecanoate with *Allobaculum*; (R)-3-hydroxybutyric acid and D-glucuronic acid with *Desulfovibrio*. As shown in Figure 12B, at 95 days of age, the positive correlation (*P* < 0.05) included between 1-aminocyclopropanecarboxylic acid with *Sphaerochaeta*; (R)-3-hydroxybutyric acid with *Clostridium*, *Streptococcus*, *02d06*, *Allobaculum*, and *Sarcina*; D-glucuronic acid with *Clostridium*, *Streptococcus*, *02d06*, *Allobaculum*, and *Sarcina*; maleic acid with *Treponema*; (+)-7-isojasmonic acid with *Lachnospira*; 8(R)-hydroperoxylinoleic acid with *Clostridium*, *Allobaculum*, and *Sarcina*; 9,10-12,13-diepoxyoctadecanoate with *L7A_E11*, *Allobaculum*, and *Sarcina*; In addition, the negative correlation (*P* < 0.05) included between N6,N6,N6-trimethyl-L-lysine with *Gemmiger*, *Allobaculum*, *Sarcina*, and *Campylobacter*; (R)-3-hydroxybutyric acid with *Treponema*; D-glucuronic acid with *Treponema* and *Pediococcus*; maleic acid with *Clostridium*, *Gemmiger*, *Blautia*, *Allobaculum*, and *Sarcina*; maleic acid with *L7A_E11* and *02d06*; 8(R)-hydroperoxylinoleic acid with *Treponema* and *Pediococcus*; 9,10-12,13-diepoxyoctadecanoate with *Treponema* and *Sphingomonas*. As shown in Figure 12C, at 125 days of age, the positive correlation (*P* < 0.05) included between N-acetylhistamine with *Phascolarctobacterium*. In addition, the negative correlation (*P* < 0.05) included between 1-aminocyclopropanecarboxylic acid with *Prevotella*; D-glucuronic acid with *Lactobacillus* and *Dorea*; 1-naphthylamine with *Parabacterioides*.

As shown in Figure 12D, at 65 days of age, the positive correlation (*P* < 0.05) included between skatole with *SMB53*, *Faecalibacterium*, and *02d06*; tryptamine with *Sphingomonas*; phenylethyamine with *RFN20*; spermidine and spermine with *Blautia*, *Faecalibacterium*, *Collinsella*, *Catenibacterium*, and *Sphingomonas*; acetate and propionate with *Gemmiger*, *Roseburia*, and *Faecalibacterium*; butyrate with *Gemmiger*, *Blautia*, *Roseburia*, *Faecalibacterium*, *Coprococcus*, *Collinsella*, and *Bulleidia*; valerate with *SMB53*; isobutyrate and isovalerate with *SMB53*, *Clostridium*, *Turicibacter*, *02d06*, and *unclassified_Erysipelotriichaceae*; total SCFA with *Gemmiger*, *Roseburia*, *Faecalibacterium*, *Coprococcus*, and *Bulleidia*. In addition, the negative correlation (*P* < 0.05) included between indole with *Roseburia* and *Corprococcus*; skatole with *Dorea*; tryptamine with *SMB53*, *Turicibacter*, *02d06*, *unclassified_Erysipelotriichaceae*, and *Phascolarctobacterium*; cadaverine with *Turicibacter* and *02d06*; 1,7-heptanediamine with *02d06* and *unclassified_Erysipelotriichaceae*; tyramine with *Clostridium* and *Turicibacter*; spermidine and spermine with *Treponema*, *02d06*, and *Parabacteroides*; butyrate with *Treponema*; valerate with *Sphaerochaeta*; isobutyrate and isovalerate with *Dorea*.

As shown in Figure 12E, at 95 days of age, the positive correlation (*P* < 0.05) included between indole with *Anaerovibrio*; skatole with *Lachnospira* and *Anaerovibrio*; tryptamine with *Clostridium*, *Gemmiger*, *Blautia*, and *Bulleidia*; phenythylamine with *Ruminococcus*, *Clostridium*, *Gemmiger*, *Allobaculum*, and *Sarcina*; putrescine with *Ruminococcus*, *Gemmiger*, and *Bulleidia*; cadaverine with *Bacteroides* and *Sphaerochaeta*; 1,7-heptanediamine with *Gemmiger*, *All-obaculum*, and *Campylobacter*; tyramine with *Gemmiger* and *Bacteroides*; spermidine with *Clostridium*, *Gemmiger*, *Blautia*, *Allobaculum*, and *Sarcina*; spermine with *Lactobacillus* and *Gemmiger*; acetate, propionate, butyrate, and total SCFA with *Roseburia*, *Lachnospira*, and *Faecalibacterium*; butyrate with *Clostridium*; valerate and isobutyrate with *Lachnospira*; isovalerate with *Lachnospira*, *Phascolarctobacterium*, and *Anaerovibrio*. In addition, the negative correlation (*P* < 0.05) included between skatole with *Ruminococcus*; tryptamine and phenythylamine with *Phascolarctobacterium*; putrescine with *L7A_E11* and *Phascolarctobacterium*; cadaverine with *Bacteroides*, *Sphaerochaeta*, and *L7A_E11*; 1,7-heptanediamine with *Trepnema*; tyramine with *L7A_E11* and *Phascolarctobacterium*; butyrate with *Streptococcus*; isobutyrate and valerate with *Akkermansia*.

As shown in Figure 12F, at 125 days of age, the positive correlation (*P* < 0.05) included between indole with *Lactobacillus* and *Streptococcus*; tryptamine with *SMB53*, *Gemmiger*, *Blautia*, and *Faecalibacterium*; phenylethylamine with *Coprococcus*; putrescine with *Gemmiger*, *Blautia*, and *Faecalibacterium*; cadaverine with *Gemmiger* and *Blautia*; tyramine with *Gemmiger*; spermidine with *SMB53* and *Blautia*; acetate with *Gemmiger*, *Faecalibacterium*, and *Lachnospira*; propionate with *Gemmiger* and *Faecalibacterium*; butyrate with *Gemmiger*, *Roseburia*, *Blautia*, and *Faecalibacterium*; valerate with *Gemmiger*, *[Prevotella]*, and *Lachnospira*; isobutyrate and isovalerate with *Gemmiger* and *Lachnospira*; total SCFA with *Gemmiger*, *Faecalibacterium*, and *Lachnospira*. In addition, the negative correlation (*P* < 0.05) included between skatole with *Parabacteroides*; tryptamine with *Oscillospira*; phenylethylamine with *Prevotella* and *Phascolarctobacterium*; putrescine with *Oscillospira*, *Bacteroides*, and *Dehalobacterium*; cadaverine with *Oscillospira*, *Phascolarctobacterium*, *Bacteroides*, and *Dehalobacterium*; tyramine with *Bacteroides*; spermidine with *Oscillospira*; acetate with *Phascolarctobacterium* and *Bacteroides*; propionate with *Oscillospira*, *02d06*, and *Bacteroides*; butyrate with *Oscillospira* and *Bacteroides*; total SCFA with *02d06* and *Bacteroides*.

## Discussion

The importance of gut microbiota is widely acknowledged because of their pivotal role in the health of animals, whose diversity provides the host with beneficial functions (Kim and Isaacson, 2015). Probiotics and synbiotics play prodigious roles in regulating the gut microbiota and the metabolites of the host. Moreover, probiotics and synbiotics addition to sows’ diets may be envisaged as beneficial for sows and their progeny. The results of the present study clearly show that probiotics and synbiotics supplementation in sows’ diets results in a marked difference in the composition and metabolic capacity of the colonic microbiota in the offspring. These findings provide a theoretical basis for the application of probiotics and synbiotics to the “integration of mother-offspring” regulation.

The colon is the main site of microbial colonization, which plays a key role in animal health (Luo et al., 2013). In general, high microbial diversity is favorable for the overall health and productivity of animals (Hildebrand et al., 2013). In the present study, maternal probiotics and synbiotics addition did not affect the richness and diversity of colonic microbiota in the offspring pigs. This is consistent with the results of Zhang et al. (2016), who reported that oral administration of *Lactobacillus* did not change the α-diversity of cecal and colonic microbiota. In addition, the β-diversity analysis indicated that the four groups had discrete microbiota structures at 65 days of age, as evidenced by the Bray-Curtis distance. The PLS-DA also showed that the colonic microbiota structure of different groups at 65, 95, and 125 days of age were distinct, suggesting that the offspring pigs showed different microbiota compositions due to probiotics and synbiotics addition to sows’ diets.

Bacteria belonging to Firmicutes, Bacteroidetes, and Proteobacteria phyla constitute the dominant microbiota in porcine intestines (McCormack et al., 2017). In the present study, the most predominant phyla identified in colonic contents were Firmicutes and Bacteroidetes at 65, 95, and 125 days of age, which accounted for more than 90% of microbiota. This is in accordance with previous studies showing that Firmicutes and Bacteroidetes are the most dominant phyla in pigs (Niu et al., 2015; Chae et al., 2016).

Probiotics and synbiotics are commonly used as feed additives to regulate the gut microbiota community. In the present study, maternal probiotics and synbiotics addition altered the colonic microbiota community of offspring pigs. Our analysis showed that the Actinobacteria abundance in the SP group was higher at 65 days of age. Previous studies demonstrated that yeast supplementation resulted in the increased abundance of Actinobacteria, which is associated with its beneficial effects of maintaining the homeostasis of intestinal microbiota (Kiros et al., 2018). In addition, the abundances of *Blautia* and *Catenibacterium* were increased and the ratio of feed/gain was decreased with no significance in the SP group at 65 days of age (Zhu et al., 2022). These findings are consistent with a previous study that reported that *Blautia* and *Catenibacterium* had a positive association with feed efficiency (Bergamaschi et al., 2020). Furthermore, at 95 days of age, maternal probiotics addition increased the abundances of several beneficial bacteria, such as Tenericutes, *Clostridium*, *Gemmiger*, *Blautia*, and *Roseburia.* These are consistent with the results of LEfSe analysis, which showed that *Clostridum*, *Gemmiger*, *Roseburia*, *Allobaculum*, and *Sarcina* were more abundant in the SP group in the present study. Previous studies reported that *Blautia* and *Roseburia* play an important regulatory role in lipid metabolism and fat deposition (Kasahara et al., 2018; Ozato et al., 2019). *Clostridium* spp. contributes to complex carbohydrate breakdown in the gut and produces SCFAs, which are beneficial to intestinal epithelial cells (El Kaoutari et al., 2013). *Gemmiger*, *Faecalibacterium*, *Bulleidia*, and *Prevotella* were the core functional genera after *Lactobacillus* supplementation (Zhang et al., 2021). Moreover, the supplementation with probiotics increased the abundances of beneficial bacteria in the colonic mucosa, including *Prevotella*, *Faecalibacterium*, *Gemmiger*, and *Coprococcus* (Qian et al., 2020). Therefore, these findings demonstrate that maternal probiotics addition may improve the lipid metabolism of offspring and maintain the homeostasis of intestinal microbiota by increasing beneficial microbiota.

Previous studies demonstrated that the health-associated lactic acid bacteria, such as *Lactobacillus* spp., play important roles in preventing disease (Gresse et al., 2017). Therefore, it is commonly used as probiotics to maintain the homeostasis of gut microbiota. In addition, some species of *Lactobacillus* help to shape the composition of the gut microbiota by producing antimicrobial bacteriocins (Zhang et al., 2016). At the genus level, *Lactobacillus* was dominant at 65, 95, and 125 days of age in the present study. Previous studies demonstrated that *Treponema* could cause colon inflammation and swine blood dysentery (Mølbak et al., 2006). Maternal probiotics addition decreased the relative abundance of *Treponema* at 95 days of age in the present study, suggesting that probiotics inhibited the growth of harmful bacteria. Moreover, a previous study also demonstrated that *Lactobacillus* could decrease the abundance of *Treponema* (Kim and Isaacson, 2015). These findings suggest that maternal probiotics addition inhibited several potentially harmful bacteria.

Spirochaetes include several pathogens, such as *Treponema* (Nakamura, 2020). Proteobacteria also contain opportunistic pathogens, such as *Campylobacter*, *Escherichia*, *Shigella*, *Salmonella*, and *Helicobacter* (Khan and Chousalkar, 2021), and the proliferation of multiple anaerobic bacteria in Proteobacteria can lead to gut dysbiosis and inflammation in the host (Bäumler and Sperandio, 2016). In addition, *Streptococcus* is a potentially pathogenic bacteria, being presumably involved in colon carcinogenesis (Goyette-Desjardins et al., 2014). In the present study, the abundances of these harmful bacteria, including Spirochaetes, Proteobacteria, *Treponema*, *Streptococcus*, *Campylobacter*, *Staphylococcus*, Acidobacteria, Gemmatimonadetes, and Chloroflexi were increased in the SA group. These findings suggest that maternal antibiotic addition exerted detrimental effects on the gut of offspring pigs through an increase of potentially deleterious bacterial species. Moreover, a previous study also demonstrated that antibiotic alters the nutritional landscape of the gut and leads to the expansion of pathogenic populations (Bäumler and Sperandio, 2016).

The gut microbiota is a vital regulator of host metabolism (Schoeler and Caesar, 2019). PICRUSt2 was used to predict putative metagenomes based on 16S rRNA gene profiles and to determine the metabolic functional changes. In the present study, the majority of pathways were carbohydrate metabolism, amino acid metabolism, metabolism of cofactors and vitamins, and metabolism of terpenoids and polyketides. The gut microbiota not only participates in the metabolism of carbohydrate and amino acid but also in the production of vitamin (Bik, 2009). In the present study, pathways related to pantothenate and CoA biosynthesis, ascorbate and aldarate metabolism, as well as phosphonate and phosphinate metabolism were enriched in the SS group, suggesting that maternal synbiotics addition increased the metabolism of cofactors and vitamins, carbohydrate metabolism, and metabolism of other amino acids. Consistent with these results, pathway enrichment analysis in metabolomics confirmed the effects of dietary probiotics and synbiotics addition on the metabolism.

In addition, our results showed that the pathways belonging to the xenobiotics biodegradation and metabolism were enriched in the SA group at 65 and 95 days of age, suggesting that maternal antibiotic addition promotes the metabolism of xenobiotics of offspring pigs. The study on germ-free animals and conventional animals in which the intestinal microbiota composition is affected by treatment with antibiotics or dietary modification indicated that the gut microbiota is involved in xenobiotic metabolism (Koppel et al., 2017). This may be related to the negative effects of maternal antibiotic addition on the offspring. Several studies demonstrated that maternal antibiotic exposure during pregnancy increases the risk of childhood allergic diseases and results in the dysbiosis of gut microbiota (Zhong et al., 2020). However, the underlying mechanisms require further investigation.

Gut microbiota and its metabolites impact physiology function and modulate metabolic activities of the host (Schroeder and Bäckhed, 2016). Furthermore, these metabolites are key intermediates in host-microbiota interactions and influence a wide variety of physiological functions (Koh and Bäckhed, 2020). Colonic metabolites can reflect the results of nutrient metabolism by both the gut bacteria and the host. In the present study, PCA and OPLS-DA analyses showed a clear separation of colonic metabolites profiles of offspring pigs due to maternal probiotics and synbiotics addition. Moreover, the heatmap of different metabolites were also distinct at different stages. These findings suggest that maternal probiotics and synbiotics addition has a significant effect on the metabolic profiles of offspring. Our results also showed that the different metabolites mainly belong to amino acid, carbohydrate, and lipid. These may result from the fact that gut microbiota may affect the metabolism of these compounds (Wang et al., 2020). When VIP > 2, the different metabolites were beta-sitosterol, trioxilin A3, D-glucuronic acid, and 9,10–12,13-diepoxyoctadecanoate at 65 days of age, as well as phytosphingosin and N6,N6,N6-trimethyl-L-lysine at 95 days of age, and tetracosanoic acid, N-acetylhistamine, palmitic acid, fructose 6-phosphate, putrescine, and chenodeoxycholic acid at 125 days of age. These changes may have beneficial effects on the offspring. For example, beta-sitosterol exhibits anti-inflammatory activity in intestinal endothelial cells (Loizou et al., 2010), putrescine is essential for the proliferation of intestinal epithelial cells (Mouillé et al., 2003), and hydroxycinnamic acids could inhibit intestinal pathogens (Lee et al., 2006).

Regarding the specific case of the putrescine, spermidine, and spermine, of which colonic concentrations were increased in the offspring obtained from sows supplemented with either probiotics or synbiotics, it is worth noting that polyamines are known to play important roles in the intestinal epithelium, and more largely on the intestinal mucosa physiology. Indeed, these polyamines are involved in fluid secretion by colonic crypts (Cheng et al., 2004), and in post-prandial colonic motility (Fioramonti et al., 1994). Dietary supplementation with spermidine reinforces the intestinal barrier function in mice (Ma et al., 2020d), and putrescine stimulates DNA synthesis in intestinal epithelial cells (Ginty and Seidel, 1989). In addition, polyamines appear required for intestinal epithelium renewal (McCormack et al., 1993; Wang, 2007; Rao et al., 2020). Of note, a mixture of putrescine, spermidine, and spermine has been found to be necessary for normal postnatal development of the small intestine and colon mucosa (Löser et al., 1999). Microbial putrescine represents a stimulant for the proliferation of colonic epithelial cells (Nakamura et al., 2021). Lastly, putrescine, spermine, and spermidine improve the integrity of the gut by increasing tight junction protein expression and mucus secretion (Oliphant and Allen-Vercoe, 2019).

The gut microbiota is able to produce putrescine, cadaverine, tyramine, and histamine from their respective amino acid precursors that are ornithine/arginine, lysine, tyrosine, and histidine, respectively (Davila et al., 2013). Tryptamine plays a role in regulating intestinal motility and immune function (Gao et al., 2018). Since the present study showed that the concentrations of putrescine, spermine, and spermidine increased in the SA, SP, and SS groups, as well as the tryptamine in the SP and SS groups, these results suggest that maternal addition with these additives could enhance the metabolism of amino acids and have beneficial effects of gut integrity. In addition, the correlation analysis showed that colonic putrescine, spermine, and spermidine concentrations were positively correlated with the abundance of several beneficial bacteria, such as *Gemmiger*, *Blautia*, and *Faecalibacterium*. This could explain the increase in colonic polyamine concentration because dietary probiotics can increase the polyamine concentration in the intestinal lumen. Therefore, there is an increasing interest in the use of gut commensal bacteria as potential probiotics, such as *Bacteroides*, *Clostridium*, *Bifidobacterium*, and *Faecalibacterium* (Tofalo et al., 2019).

Palmitic acid was reported to damage gut epithelium integrity (Carta et al., 2017) and initiate inflammatory cytokine production (Ghezzal et al., 2020). In the present study, the normalized intensity of palmitic acid was decreased by maternal probiotics and synbiotics addition, which may be beneficial to the gut health of the host. (R)-3-hydroxybutyric acid is a key metabolite of butanoate metabolism, which plays an important role in regulating intestinal immune tolerance to antigens (Zou et al., 2021). In the present study, (R)-3-hydroxybutyric acid was increased in the SP and SS groups, suggesting the beneficial effects of maternal probiotics and synbiotics addition on the gut health of offspring. These may be related to the changes in gut microbiota. (R)-3-hydroxybutyric acid is synthesized *via* the metabolism of butyrate and acetate (Murugesan et al., 2018). In the present study, the correlation analysis between the metabolites and microbiota showed that (R)-3-hydroxybutyric acid was positively associated with several SCFAs-producing bacteria, including *Faecalibacterium*, *Blautia*, *Clostridium*, and *Streptococcus*. A previous study also reported that *Bacteroides*, *Bifidobacterium*, *Prevotella*, *Ruminococcus*, *Blautia*, *Clostridium*, and *Streptococcus* can produce acetate, and *Coprococcus*, *Faecalibacterium*, and *Roseburia* can produce butyrate (Murugesan et al., 2018). These findings suggest that the addition of probiotics and synbiotics could alter the metabolite profiles by affecting the composition of gut microbiota.

Indole and skatole are the main end-products of tryptophan metabolism by gut microbiota (Lu et al., 2021). Indole can improve the integrity of colon barrier function, while skatole can cause gut epithelial cell dysfunction (Gao et al., 2018). Indeed, skatole in excess can induce cell death in colonocytes (Kurata et al., 2019). In the present study, the colonic indole concentration increased and skatole decreased in the SS group, suggesting that sow dietary synbiotics addition has a beneficial effect on the gut barrier function and gut epithelial cell function. However, maternal probiotics addition results in the changes of colonic indole and skatole concentrations that need to be further studied since gut health can be affected by several bacterial metabolites present in the colonic fluid. Antibiotic use is an important factor influencing skatole level (Gao et al., 2018). Our findings showed that the colonic concentrations of indole and skatole were increased in the SA group. However, the underlying mechanisms require further investigation.

The SCFAs, especially acetate, propionate, and butyrate, have been shown to prevent intestinal oxidative stress and inflammation and to protect the intestinal barrier (Liu et al., 2021). The butyrate is a major energy source for the colonocytes and exerts antimicrobial and anti-inflammatory activities by several mechanisms (Waldecker et al., 2008). The present study observed a significant increase in the colonic acetate and butyrate concentrations in the SP group at 65 and 125 days of age, suggesting the beneficial effects of maternal probiotics addition on gut health. Previous studies demonstrated that probiotics could accelerate the degradation of polysaccharides, thereby increasing the butyric acid concentration in the colon (Liu et al., 2014). In addition, maternal probiotics and synbiotics addition decreased the colonic SCFAs concentration at 95 days of age, which may result from the rapid absorption through the colonic epithelium by the host or utilization by other members of the gut microbiota (Loh et al., 2006). This decrease may be also related to the fact that the amount and relative proportion of each SCFA depends on the diet, microbiota composition, and expression of dedicated transporters (Macfarlane and Macfarlane, 2003). *Roseburia* produces acetate and butyrate, and *Faecalibacterium* produces butyrate (Oliphant and Allen-Vercoe, 2019). The correlation analysis showed that the colonic acetate, propionate, and butyrate concentrations were positively correlated with *Gemmiger*, *Roseburia*, and *Faecalibacterium* abundances in the present study. Moreover, the abundances of *Roseburia* and *Faecalibacterium* were increased in the SP group at 65 days of age, whereas decreased in the SS group at 95 days of age. This may be the reason why maternal probiotics addition increased the colonic butyrate concentration, whereas synbiotics addition decreased the acetate and butyrate concentrations, such a decrease being presumably deleterious given notably the role of these metabolites for energy supply in colonocytes (Leschelle et al., 2000).

## Conclusion

**FIGURE 13:**
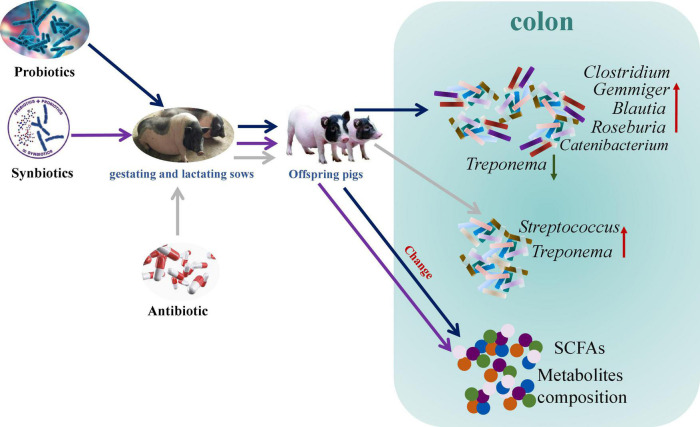
Schematic presentation of the effects of maternal probiotics and synbiotics addition to sows’ diets on colonic microbiome and metabolome of offspring pigs.

Maternal probiotics addition increased *Catenibacterium*, *Clostridium*, *Gemmiger*, *Blautia*, and *Roseburia* abundances and decreased *Treponema* abundance, whereas antibiotic addition increased *Treponema* and *Streptococcus* abundances in the colon of offspring pigs. In addition, maternal probiotics and synbiotics addition affected the metabolism pathways, including carbohydrate and amino acid metabolism, as well as cofactors and vitamins metabolism. These findings suggest that maternal probiotics and synbiotics addition present beneficial effects on gut health by altering the gut microbiota composition, their metabolites, and metabolic functions of the gut microbiota (Figure 13). Our results provide insights into the consequences of an intervention on the maternal gut microbiota for the offspring microbiota composition and metabolic activity.

## Data availability statement

The datasets presented in this study can be found in online repositories. The names of the repository/repositories and accession number(s) can be found in the article/Supplementary material.

## Ethics statement

The animal study was reviewed and approved by the Animal Care and Use Committee of Institute of Subtropical Agriculture, Chinese Academy of Sciences.

## Author contributions

XK and YY conceived and designed the experiments and revised the manuscript. QZ analyzed the data and wrote the manuscript. QZ and MS completed the feeding experiments and analyses. QZ, MS, YC, YTL, and YL assisted in the completion of part of the feeding experiments and sample collection. QZ, FB, and MA amended the manuscript. All authors read and approved the final manuscript.

## Footnotes

1
https://docs.qiime2.org/2019.4/tutorials/

2
https://www.r-project.org/

3
http://huttenhower.sph.harvard.edu/galaxy/

4
https://www.ncbi.nlm.nih.gov/sra/PRJNA825463/

5
www.bioconductor.org

6
http://www.mzcloud.org/

7
mona.fiehnlab.ucdavis.edu

8
www.kegg.jp
