# Supplementary material for: Probiotics or synbiotics addition to sows’ diets alters colonic microbiome composition and metabolome profiles of offspring pigs
Source: Front Microbiol. 2022 Aug 17;13:934890. doi: 10.3389/fmicb.2022.934890 (PMC9428521; doi:10.3389/fmicb.2022.934890)
Supplement: Supplementary file 1 [file Data_Sheet_1.docx]

**Probiotics or Synbiotics Addition to Sows’ Diets Alters Colonic** **Microbiome Composition and Metabolome Profiles of Offspring Pigs**

**Qian Zhu^1,2^, Mingtong Song^1^, Md. Abul Kalam Azad^1^, Yating Cheng^1,2^, Yating Liu^1,2^, Yang Liu^1^,** **François Blachier^4^, Yulong Yin^1,2^, Xiangfeng Kong^1,2,3*^**

^1^ Hunan Provincial Key Laboratory of Animal Nutritional Physiology and Metabolic Process, Key Laboratory of Agro-ecological Processes in Subtropical Region, National Engineering Laboratory for Pollution Control and Waste Utilization in Livestock and Poultry Production, Institute of Subtropical Agriculture, Chinese Academy of Sciences, Changsha 410125, China.

^2^ College of Advanced Agricultural Sciences, University of Chinese Academy of Sciences, Beijing 100049, China.

^3^ Université Paris-Saclay, AgroParisTech, INRAE, UMR PNCA, 75005 Paris, France.

^4^ Research Center of Mini-Pig, Huanjiang Observation and Research Station for Karst Ecosystems, Chinese Academy of Sciences, Huanjiang 547100, China.

***Correspondence**:

Xiangfeng Kong, E-mail: nnkxf@isa.ac.cn


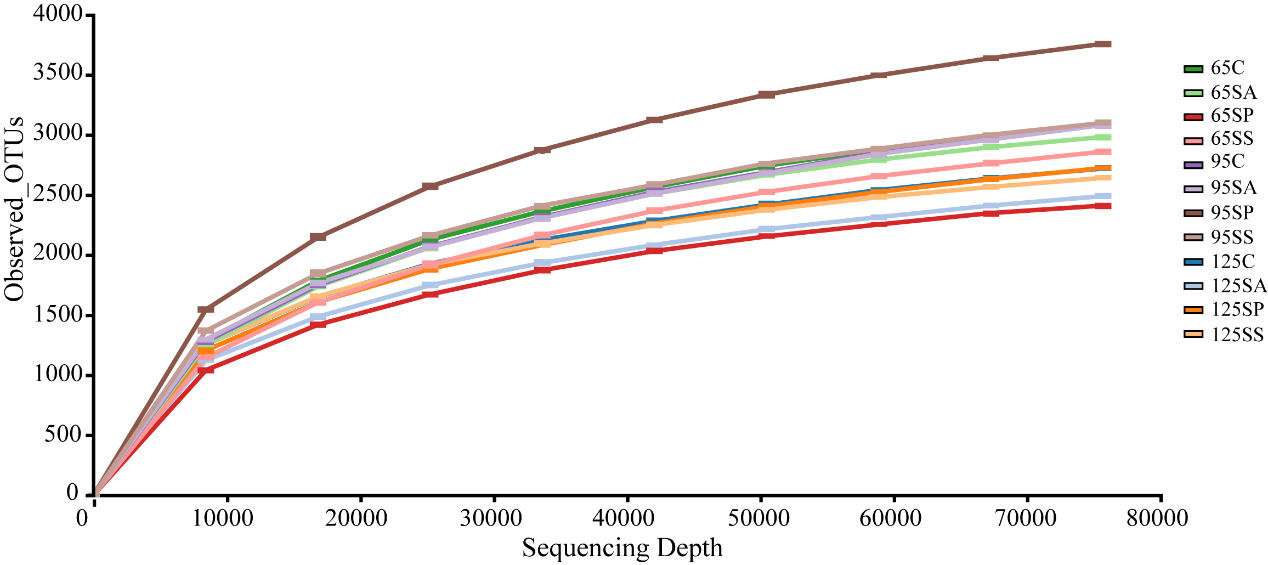


**Supplementary Figure 1** Rarefaction curves comparing the number of sequences with the number of phylotypes found in the 16S rRNA gene libraries from the microbiota in the colonic contents of offspring pigs at 65, 95, and 125 days of age. 65C, 95C, and 125C, sow fed with basal diet; 65SA, 95SA, and 125SA, sow fed with antibiotic; 65SP, 95SP, and 125SP, sow fed with probiotics; 65SS, 95SS, and 125SS, sow fed with synbiotics.


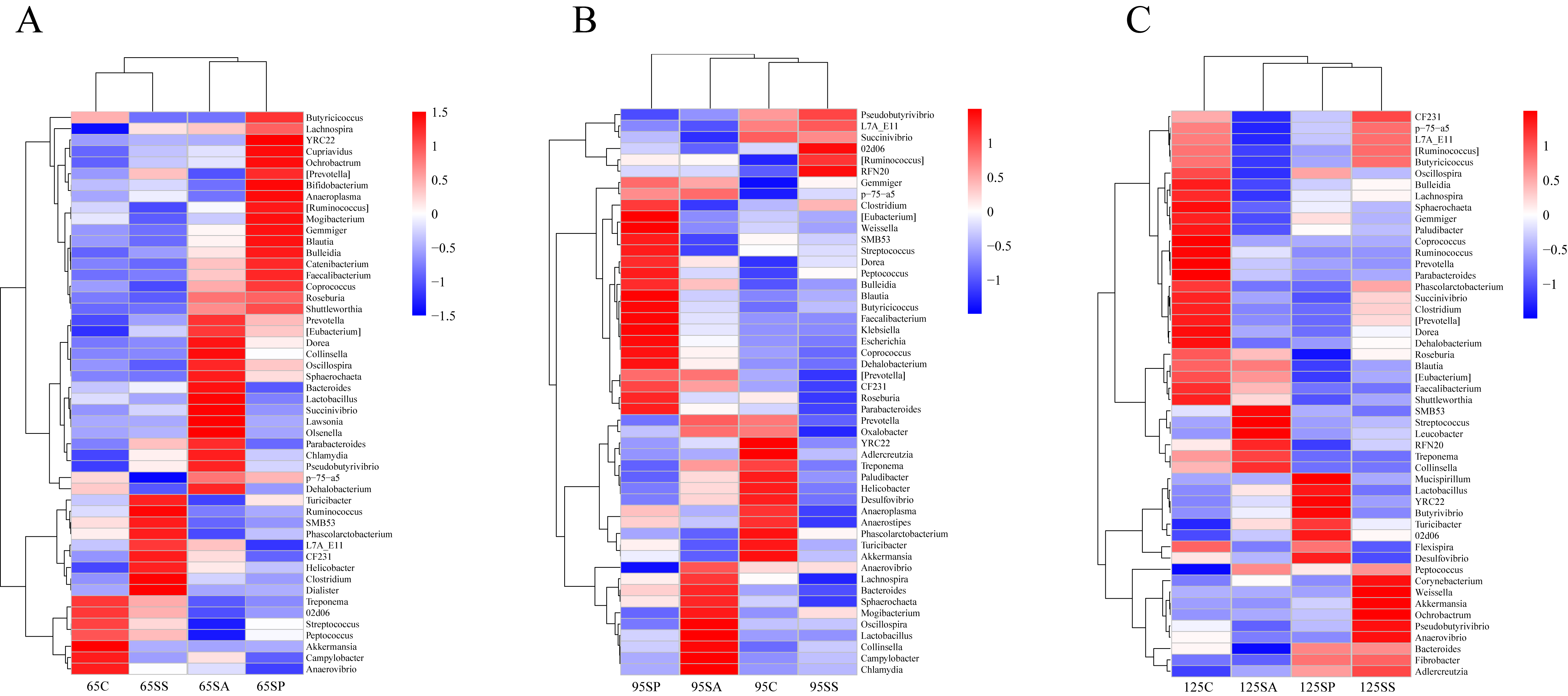


**Supplementary Figure 2** Hierarchical clustering and heatmap of microbiota taxonomic composition at genus level (the top 50 genera) in the colonic contents of offspring pigs at 65 (A), 95 (B), and 125 (C) days of age. 65C, 95C, and 125C, sow fed with basal diet; 65SA, 95SA, and 125SA, sow fed with antibiotic; 65SP, 95SP, and 125SP, sow fed with probiotics; 65SS, 95SS, and 125SS, sow fed with synbiotics.


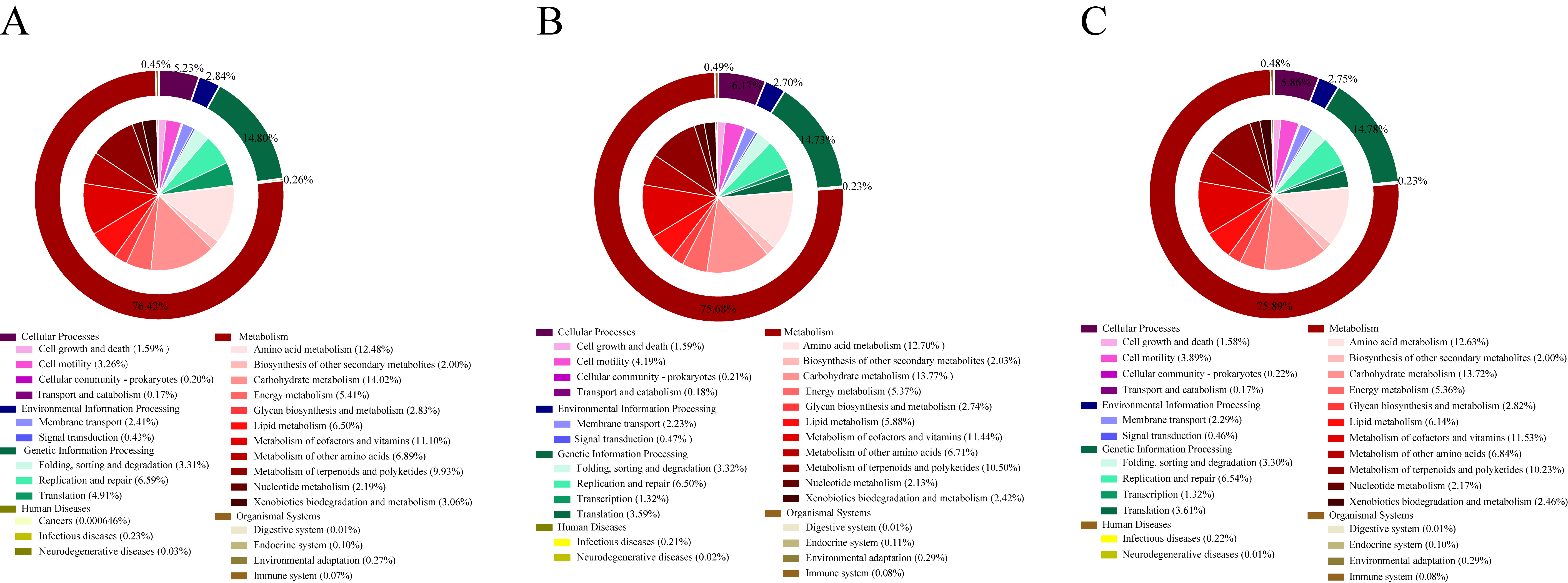


**Supplementary Figure 3** Predictive metagenomics shows the composition of functional metabolic pathways at 65 (A), 95 (B), and 125 (C) days of age using the PICRUSt2 analysis at level 1 and level 2. Different color represent different metabolic pathway at level 1 and level 2. The outer ring and inner circle around the pie chart show the relative abundance of different pathway at level 1 and level 2, respectively.


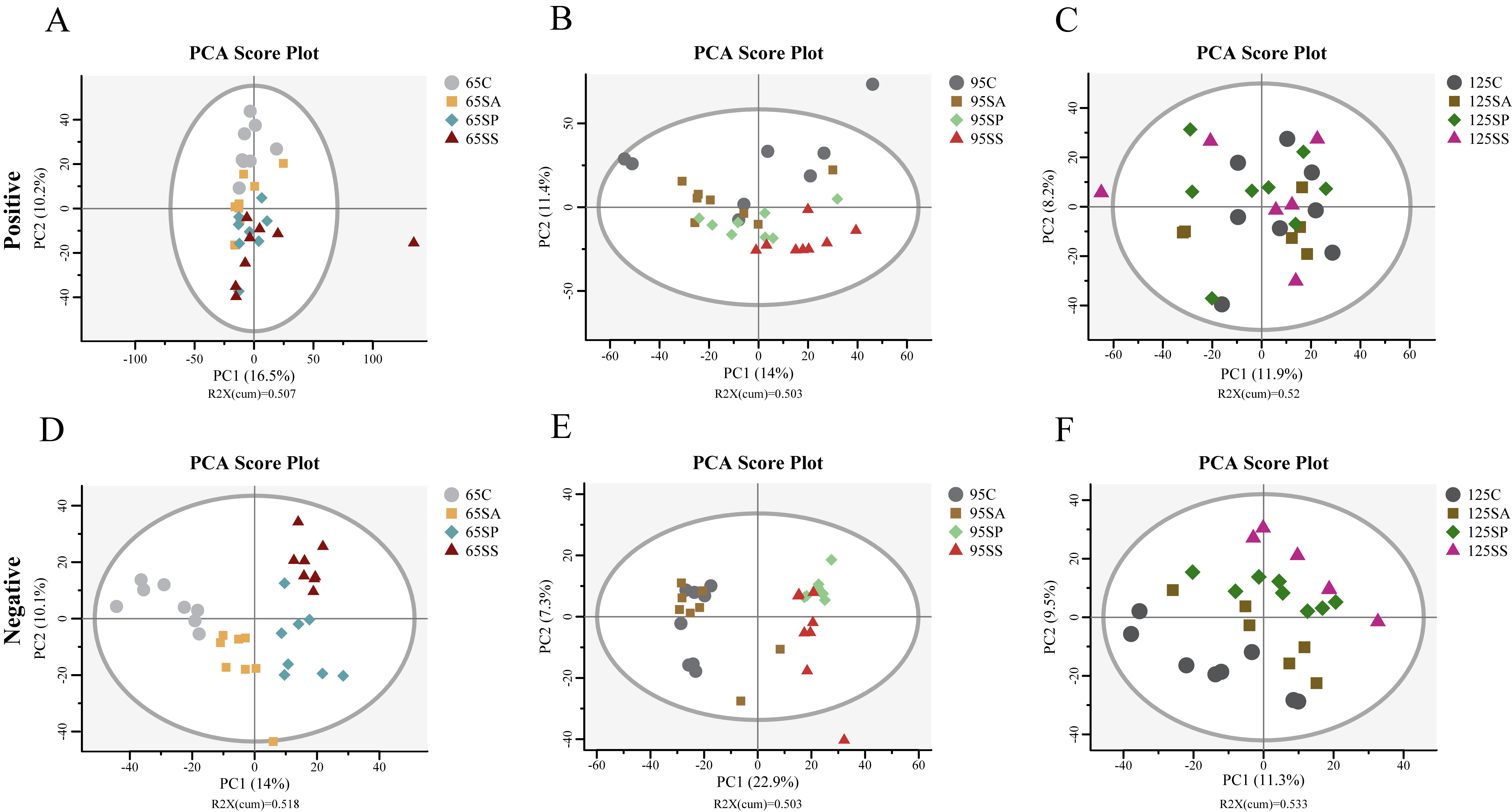


**Supplementary Figure 4** Principal component analysis (PCA) plots based on the metabolites of colonic contents in positive (A−C) and negative (D−F) models at 65, 95, and 125 days of age among the four groups. 65C, 95C, and 125C, sow fed with basal diet; 65SA, 95SA, and 125SA, sow fed with antibiotic; 65SP, 95SP, and 125SP, sow fed with probiotics; 65SS, 95SS, and 125SS, sow fed with synbiotics.


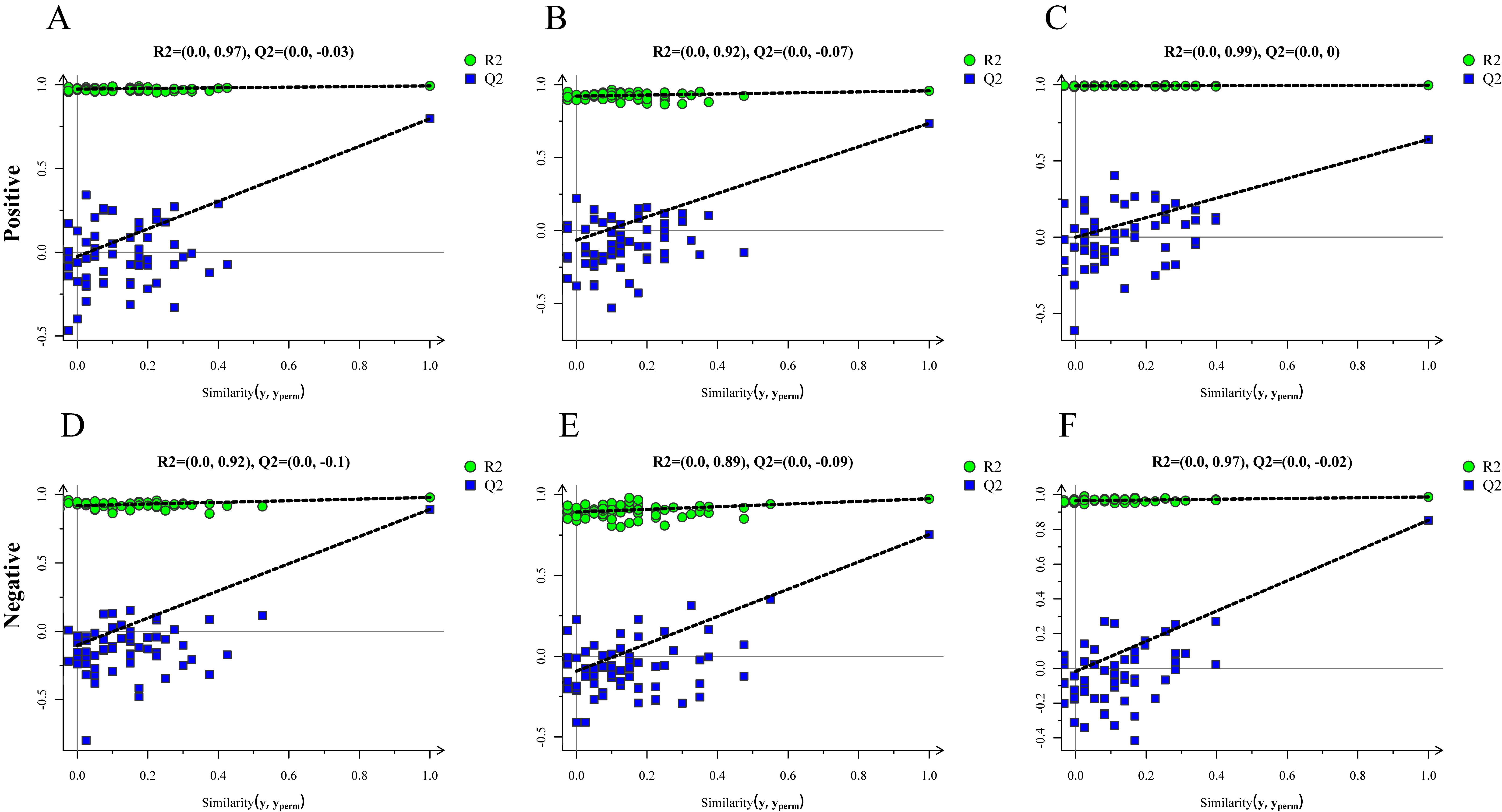


**Supplementary Figure 5** The permutations plots of orthogonal partial least squares discriminant analysis (OPLS-DA) in positive (A−C) and negative (D−F) models at 65, 95, and 125 days of age among the four groups.


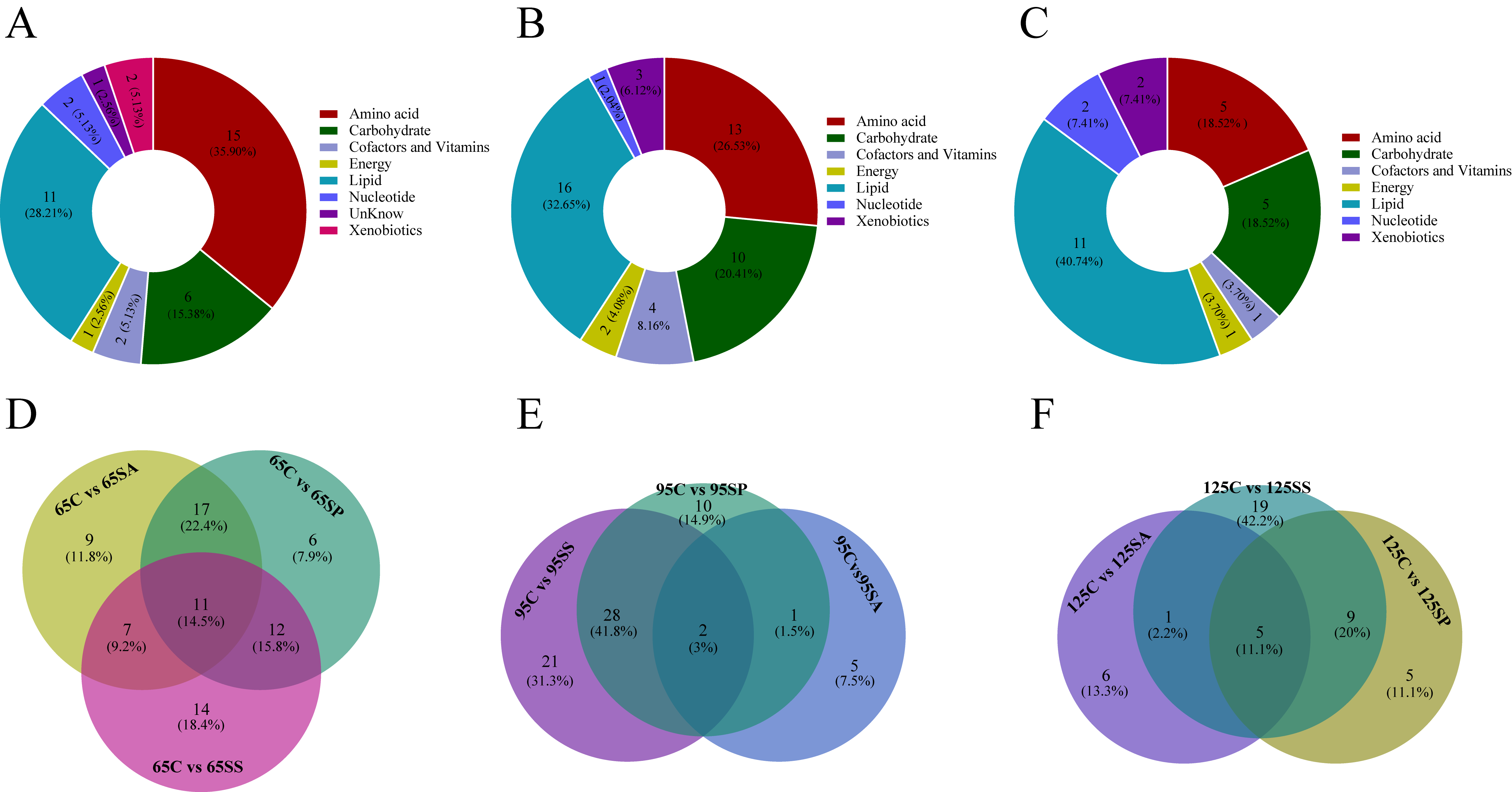


**Supplementary Figure 6** Different metabolites analysis of offspring in colonic contents. Pie chart of different metabolite classification at 65, 95, and 125 days of age (A−C). Venn diagram of different metabolites for pairwise comparison (D−F). 65C, 95C, and 125C, sow fed with basal diet; 65SA, 95SA, and 125SA, sow fed with antibiotic; 65SP, 95SP, and 125SP, sow fed with probiotics; 65SS, 95SS, and 125SS, sow fed with synbiotics.


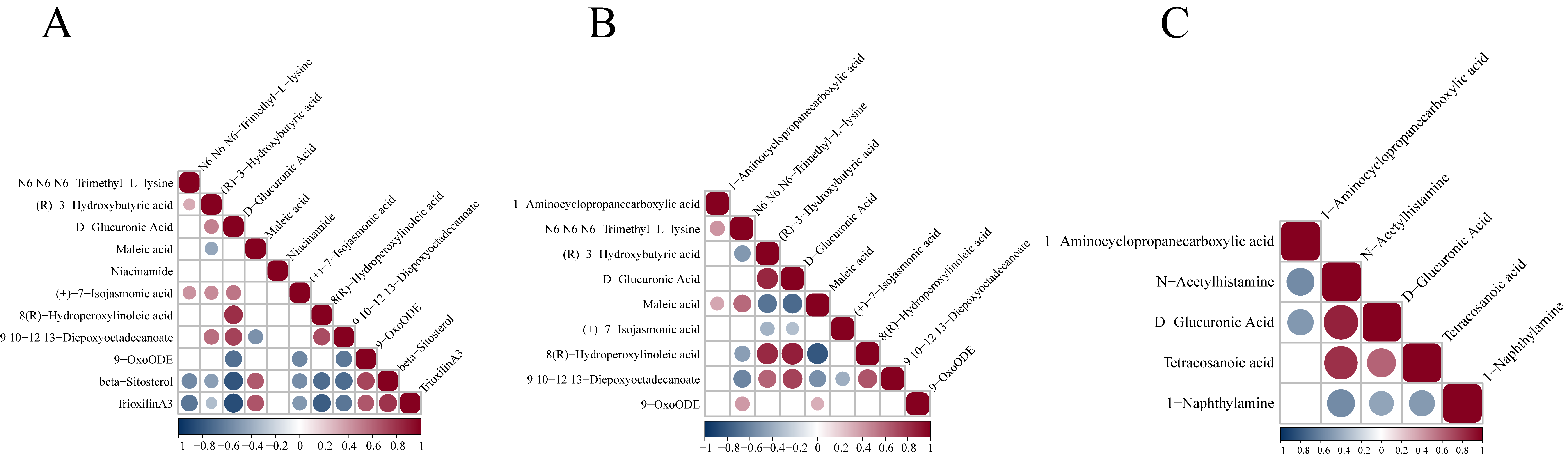


**Supplementary Figure 7** Correlation analysis of different metabolites at 65, 95, and 125 days of age (A-C). The red represents a significant positive correlation, and the blue represents a significant negative correlation.

**Supplementary Table 1** Composition and nutrient levels of basal diets for sows (air-dry basis; %).

| Items | Pregnant diet | Lactating diet |
| --- | --- | --- |
| Ingredients |  |  |
| Corn | 37.50 | 66.00 |
| Soybean meal | 9.50 | 25.00 |
| Wheat bran | 14.00 | 5.00 |
| Barley | 25.00 |  |
| Soybean hull | 10.00 |  |
| Pregnant premix^1)^ | 4.00 |  |
| Lactating premix^2)^ |  | 4.00 |
| Total | 100.00 | 100.00 |
| Nutrient levels^3)^ |  |  |
| Digestible energy (MJ/Kg) | 12.55 | 13.87 |
| Crude protein | 12.82 | 16.30 |
| Crude fiber | 4.56 | 2.87 |
| SID^4)^ Lys | 0.48 | 0.75 |
| SID Met+ Cys | 0.43 | 0.51 |
| SID Thr | 0.37 | 0.53 |
| SID Trp | 0.13 | 0.17 |
| Calcium | 0.62 | 0.65 |
| Phosphorus | 0.47 | 0.50 |

^1)^ Pregnant premix provided the following per kg of diet: CaHPO_4_⋅2H_2_O 10 g, NaCl 4 g, CuSO_4_⋅5H_2_O 80 mg, FeSO_4_ ⋅H_2_O 360 mg, ZnSO_4_⋅H_2_O 240 mg, MnSO_4_⋅H_2_O 100 mg, MgSO_4_⋅7H_2_O 1 g, 1% ICl 50 mg, 1% Na_2_SeO_3_ 36 mg, 1% CoCl_2_ 16 mg, NaHCO_3_ 1.4 g, VA 10 000 IU, VD_3_ 1 800 IU, VE 20 mg, VK_3_ 2.4 mg, VB_1_ 1.6 mg, VB_2_ 6 mg, VB_6_ 1.6 mg, VB_12_ 0.024 mg, folic acid 1.2 mg, nicotinamide 20 mg, pantothenic acid 12 mg, biotin 0.12 mg, ferrous glycinate 100 mg, choline chloride 1g, phytase 200 mg, fruity 80 mg, limestone 12 g.

^2)^ Lactating premix provided the following per kg of the diet: CaHPO_4_⋅2H_2_O 10 g, NaCl 4 g, CuSO_4_⋅5H_2_O 80 mg, FeSO_4_ ⋅H_2_O 360 mg, ZnSO_4_⋅H_2_O 240 mg, MnSO_4_⋅H_2_O 100 mg, 1% ICl 50 mg, 1% Na_2_SeO_3_ 36 mg, 1% CoCl_2_ 16 mg, NaHCO_3_ 1.4 g, VA 10 000 IU, VD_3_ 1 800 IU, VE 20 mg, VK_3_ 2.4 mg, VB_1_ 1.6 mg, VB_2_ 6 mg, VB_6_ 1.6 mg, VB_12_ 0.024 mg, folic acid 1.2 mg, nicotinamide 20 mg, pantothenic acid 12 mg, biotin 0.12 mg, Lysine 1.5 g, ferrous glycinate 100 mg, choline chloride 1g, phytase 200 mg, fruity 80 mg, limestone 12 g.

^3)^ Nutrient levels were calculated values.

^4)^ SID: standard ileum digestible.

**Supplementary Table 2** Composition and nutrient levels of basal diets for weaned Bama mini-pigs (air-dry basis; %).

| Items | Prophase diet  (35−95 days of age) | | | Anaphase diet  (96−125 days of age) |
| --- | --- | --- | --- | --- |
| Ingredients | | | | |
| Corn | | 54.92 | 58.00 | |
| Soybean meal | | 22.00 | 18.35 | |
| Wheat bran | | 10.13 | 11.35 | |
| Rice bran | | 8.95 | 8.30 | |
| Premix^1)^ | | 4.00 | 4.00 | |
| Total | | 100.00 | 100.00 | |
| Nutrient levels^2)^ | | | | |
| Digestible energy (MJ/kg) | | 13.50 | 13.42 | |
| Crude protein | | 16.13 | 14.90 | |
| Calcium | | 0.45 | 0.44 | |
| Total Phosphorus | | 0.49 | 0.49 | |
| Lys | | 1.40 | 1.30 | |
| Met + Cys | | 0.69 | 0.66 | |
| Thr | | 0.78 | 0.74 | |

^1)^ Premix provided the following per kilogram of diets: enzyme preparation (including phytase, protease, and lipase) 1.2 g, VA 26 000 IU, VD_3_ 10 000 IU, VE 70 IU, VK_3_ 10 mg, VB_1_ 10 mg, VB_2_ 25 mg, VB_6_ 10 mg, VB_12_ 0.075 mg, biotin 0.4 mg, folic acid 5 mg, nicotinamide 100 mg, pantothenic 50 mg, choline 1600 mg, flavoring agent 500 mg, edulcorant 300 mg, acidulating agent 5 g, CuSO_4_·5H_2_O 898 mg, MnSO_4_·H_2_O 298 mg, ZnSO_4_·H_2_O 600 mg, FeSO_4_·H_2_O 501 mg, Ca(IO_3_)_2_ 0.9 mg, as Na_2_SeO_3_ 0.7 mg, CoSO_4_·H_2_O 1.2 mg, glucose 2.1 g, antioxidants 0.4 g, anti-mildew agent 1 g, Ca (as CaHPO_4_ and CaCO_3_) 3.42 g, P (as CaHPO_4_ ) 1.155 g.

^2)^ Nutrient levels were calculated values.

**Supplementary Table 3** The richness and diversity of microbiota in the colonic contents of offspring at 65, 95, and 125 days of age

| Item | Control group | SA group | SP group | SS group | *P*-Value |
| --- | --- | --- | --- | --- | --- |
| OTUs | | | | | |
| 65 days of age | 4810.25±396.60 | 3852.38±496.60 | 3908.25±319.35 | 3649±346.48 | 0.19 |
| 95 days of age | 5091.88±428.24 | 5505.50±422.65 | 5913.63±368.94 | 4768.38±461.33 | 0.27 |
| 125 days of age | 4018.00±379.72 | 3461.83±254.00 | 3553.50±217.46 | 3598.33±192.04 | 0.51 |
| Chao1 | | | | | |
| 65 days of age | 3838.66±270.18 | 3006±387.85 | 2924.24±235.14 | 3010.1±269.86 | 0.11 |
| 95 days of age | 4063.46±267.73 | 4351.84±235.67 | 4748.04±281.66 | 3957.41±344.2 | 0.29 |
| 125 days of age | 4063.46±267.73 | 4351.84±235.67 | 4748.04±281.66 | 3957.41±344.2 | 0.49 |
| Observed species | | | | | |
| 65 days of age | 3319.93±231.86 | 2644.96±331.86 | 2623.29±196.65 | 2647.03±218.02 | 0.16 |
| 95 days of age | 3174.10±217.47 | 3348.65±178.70 | 3911.59±223.87 | 3272.28±247.01 | 0.14 |
| 125 days of age | 3174.10±217.47 | 3348.65±178.70 | 3911.59±223.87 | 3272.28±247.01 | 0.73 |
| Shannon | | | | | |
| 65 days of age | 7.27±0.30 | 6.84±0.44 | 7.31±0.28 | 6.66±0.29 | 0.44 |
| 95 days of age | 7.46±0.29 | 7.48±0.32 | 8.12±0.24 | 7.39±0.31 | 0.24 |
| 125 days of age | 7.46±0.29 | 7.48±0.32 | 8.12±0.24 | 7.39±0.31 | 0.31 |
| Simpson | | | | | |
| 65 days of age | 0.94±0.01 | 0.92±0.02 | 0.95±0.01 | 0.92±0.01 | 0.22 |
| 95 days of age | 0.95±0.01 | 0.93±0.02 | 0.96±0.01 | 0.92±0.02 | 0.32 |
| 125 days of age | 0.95±0.01 | 0.93±0.02 | 0.96±0.01 | 0.92±0.02 | 0.19 |
| Pielou’s | | | | | |
| 65 days of age | 0.62±0.02 | 0.61±0.03 | 0.64±0.02 | 0.59±0.02 | 0.36 |
| 95 days of age | 0.64±0.02 | 0.64±0.02 | 0.68±0.02 | 0.63±0.02 | 0.48 |
| 125 days of age | 0.64±0.02 | 0.64±0.02 | 0.68±0.02 | 0.63±0.02 | 0.22 |

Note: Data are expressed as means with the standard error of the mean (SEM). 65, 95, and 125 represent the days of age. 65, 95, and 125 days of age, *n* = 8, 8, and 6−8 per group, respectively. Control group, sow fed with basal diet; SA group, sow fed with antibiotic; SP, sow fed with probiotics; SS, sow fed with synbiotics.
